# Dissecting cricket genomes for the advancement of entomology and entomophagy

**DOI:** 10.1007/s12551-021-00924-4

**Published:** 2022-01-21

**Authors:** Kosuke Kataoka, Yuki Togawa, Ryuto Sanno, Toru Asahi, Kei Yura

**Affiliations:** 1 Comprehensive Research Organization, Waseda University, Tokyo, Japan; 2 School of Advanced Science and Engineering, Waseda University, Tokyo, Japan; 3 Graduate School of Advanced Science and Engineering, Waseda University, Tokyo, Japan; 4 Institute for Advanced Research of Biosystem Dynamics, Waseda Research Institute for Science and Engineering, Waseda University, Tokyo, Japan; 5 Research Organization for Nano & Life Innovation, Waseda University, Tokyo, Japan; 6 Computational Bio Big-Data Open Innovation Laboratory (CBBD-OIL), National Institute of Advanced Industrial Science and Technology, Tokyo, Japan; 7 Graduate School of Humanities and Sciences, Ochanomizu University, Tokyo, Japan

**Keywords:** Insecta, Polyneoptera, Orthoptera, Cricket, Genome, Alternative protein source

## Abstract

Significant advances in biophysical methods such as next-generation sequencing technologies have now opened the way to conduct evolutionary and applied research based on the genomic information of greatly diverse insects. Crickets belonging to Orthoptera (Insecta: Polyneoptera), one of the most flourishing groups of insects, have contributed to the development of multiple scientific fields including developmental biology and neuroscience and have been attractive targets in evolutionary ecology for their diverse ecological niches. In addition, crickets have recently gained recognition as food and feed. However, the genomic information underlying their biological basis and application research toward breeding is currently underrepresented. In this review, we summarize the progress of genomics of crickets. First, we outline the phylogenetic position of crickets in insects and then introduce recent studies on cricket genomics and transcriptomics in a variety of fields. Furthermore, we present findings from our analysis of polyneopteran genomes, with a particular focus on their large genome sizes, chromosome number, and repetitive sequences. Finally, how the cricket genome can be beneficial to the food industry is discussed. This review is expected to enhance greater recognition of how important the cricket genomes are to the multiple biological fields and how basic research based on cricket genome information can contribute to tackling global food security.

**Supplementary Information:**

The online version contains supplementary material available at 10.1007/s12551-021-00924-4.

## Introduction

Insects are the most prosperous organisms on the planet, and their biological diversity has attracted researchers in a variety of fields to study their mysteries. Gryllidea (crickets) has been studied for understanding developmental biology (Donoughe and Extavour 2016), regeneration biology (Nakamura et al. 2008), neuroscience (Matsumoto et al. 2018), chronobiology (Tomioka 2014), and the biophysical aspects of acoustic signaling (Song et al. 2020). For developmental biology, cricket has been used for studying embryogenesis as an alternative model that represents the insect ancestor much better than fruit fly *Drosophila melanogaster* due to its evolutionary closeness (Donoughe and Extavour 2016). In the biology of regeneration, cricket nymphs have been used as models for studying tissue and organ regeneration mechanisms, thanks to the remarkable regenerative capacity of their legs (Nakamura et al. 2008). For neuroscience, crickets have been used to study the molecular mechanisms of long-term and short-term memory formation, and it has become clear that the mechanisms of memory formation in crickets share a certain degree of similarity to those in mammals (Matsumoto et al. 2018). The findings of the studies described here have been translated to the molecular level by the nature of the cricket, which is readily available for reverse genetic techniques, including RNA interference (RNAi) (Mito and Noji 2008). The collection of these studies has allowed crickets to be used as model organisms that best represent the insect ancestor and has led to the sophistication of protocols in the fields of molecular biology, developmental biology, behavior, and neuroscience.

Moreover, the order Orthoptera, to which crickets belong, has diversified into a number of lineages, occupy all possible terrestrial habitats except for the polar regions, and play an important role in their ecosystems (Song 2018). In addition to obvious habitats such as temperate grasslands and tropical rainforests, many orthopteran insects inhabit unexpected environments. For example, the family Gryllotalpidae, commonly known as mole crickets, have adapted to a true subterranean lifestyle (Frank and Parkman 1999). The mole cricket’s paws are optimized for burrowing, and they spend most of their life underground. Additionally, the family Myrmecophilidae (ant-loving crickets) inhabit ant nests with ants, which would be attacked by ants in the case of other insects (Komatsu et al. 2008). Moreover, several species in crickets are adapted to aquatic habitats. For instance, mangrove crickets *Apteronemobius asahinai*, which inhabit mangrove forest floors only during low tide, show a clear endogenous rhythm synchronized with the tidal cycle to escape submergence (Satoh et al. 2008). Other orthopteran insects that are not crickets include species that specialize in alpine, desert, and cave habitats (Dirsh 1954; Knowles 2001; Allegrucci et al. 2010).

In addition to the biological characteristics mentioned above, orthopteran insects, of which crickets are a prime example, have an important social value. Crickets are currently being seen as a potential savior for the upcoming food crisis. The world population is growing rapidly and is projected to reach 9.74 billion by 2050 (Godfray et al. 2010). To meet this population growth, urgent interventions are needed to increase food production to satisfy the growing demand (Godfray et al. 2010). The current style of food production may not be able to meet the demand because the expansion of essential land for farming is unexpected (Nellemann et al. 2009). The lack of land is exacerbated by climate changes, water scarcity, and poverty (Nellemann et al. 2009). There is, therefore, a need for a shift to alternative and new food production systems that are cheaper, more environmentally friendly, adaptable to climate changes, and sustainable. One of the most promising options is entomophagy, defined as the practice of eating insects. Entomophagy is common in many areas of the world, with 2100 species of insects being consumed in over 110 countries. Insects can utilize water and food more efficiently and have lower feed requirements and higher growth efficiency than conventional livestock (Nagasaki and Defoliart 1991; Oonincx et al. 2015). For example, crickets consume six times and three times as little feed as cattle and pigs, respectively, to produce the same amount of protein (Oonincx et al. 2015). Moreover, crickets release fewer greenhouse gasses and ammonia than conventional livestock and can be raised on organic by-products such as food waste and waste from agriculture and the food industry (Cičková et al. 2015).

The biological and food application research on crickets can be substantiated by their genome sequence data. The genetic basis that gives rise to habitat diversity in crickets is grounded in genomic information, and the effect on food productivity by cricket should be based on gene repertoire in the genome. However, there is currently very little genomic data available to the public, compared to other insects. Analyzing the genomes of the related organisms (especially those belonging to the clade that has received little attention so far) can provide a complete picture of the genomic diversity of the complete tree of life in Orthoptera and provide insight into their evolution. More importantly, understanding the genome of an organism is generally useful for the domestication of that organism (Zsögön et al. 2018).

In this review, we begin with a brief overview of the phylogenetic position of crickets, followed by the uniqueness of crickets and Orthoptera genomes among insects, the current status of cricket genome research, and finally, the potential of the cricket genome for applied research.

## Phylogenetic overview of cricket

Insects are the most diverse animals on the planet, with different species living in a variety of terrestrial and aquatic habitats from the tropics to the polar regions (Song 2018). Crickets, or Gryllidea in phylogenetic terminology, belong to the cohort Polyneoptera, which is one of the major groups of winged insects that have the ability to fold their wings flat over the bodies by bending them. Within Polyneoptera, crickets are classified in a subgroup of the order Orthoptera, which is generally characterized by acoustic communications. In this section, we provide a brief overview of the systematics of crickets from higher to lower order classification.

### Polyneoptera

**Fig. 1:**
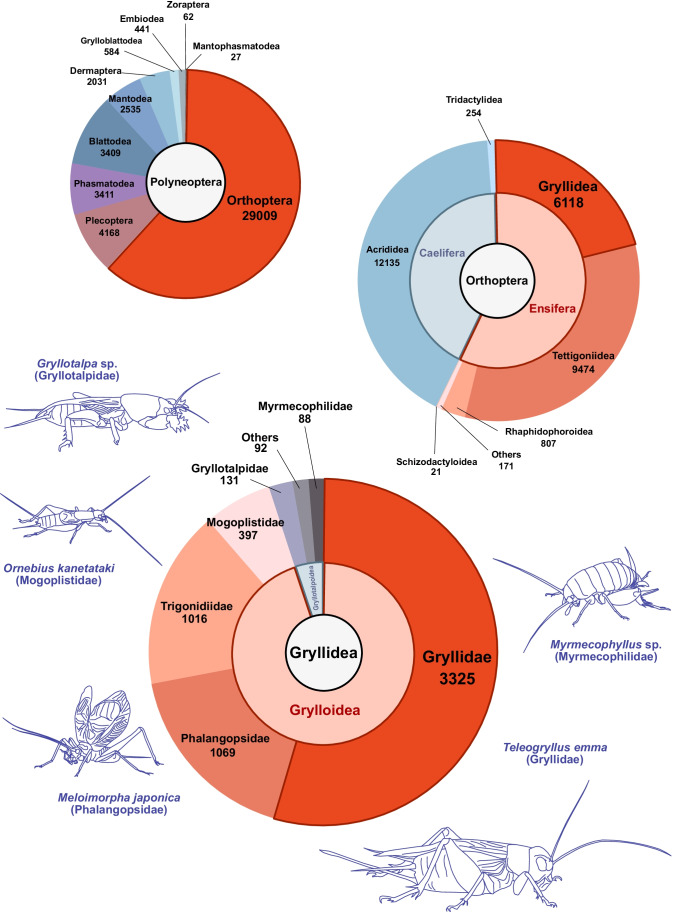
Described species of Polyneoptera, Orthoptera, and Gryllidea. The number of species shown in the pie chart represents the information available in the Orthoptera Species File as of the writing of the manuscript. Note that the Orthoptera Species File includes the superfamily Rhaphidophoroidea and Schizodactyloidea in the taxonomic groups under Ensifera, and the number of species in Tettigoniidea does not include these superfamilies

Polyneoptera comprises more than 45,000 described species in a total of ten taxonomic orders (Fig. 1). These orders comprise Orthoptera, including crickets, grasshoppers, and locusts, as well as Plecoptera (e.g., stoneflies), Phasmatodea (e.g., stick and leaf insects), Blattodea (e.g., cockroaches and termites), Mantodea (e.g., mantids), Dermaptera (e.g., earwigs), Grylloblattodea (e.g., ice crawlers), Embiodea (e.g., web-spinners), Zoraptera (e.g., angel insects), and recently described Mantophasmatodea (e.g., gladiators). Among these orders, the Orthoptera has the overwhelming number of species, accounting for more than 60% (Fig. 1) (Beccaloni 2014; Eades et al. 2014; Maehr and Hopkins 2017; Otte et al. 2017; Brock et al. 2018; Hoplins and Maehr 2018; Hopkins et al. 2018; Maehr and Hopkins 2018; Maehr et al. 2018; DeWalt et al. 2021).

Polyneopteran insects grow through incomplete or hemimetabolous metamorphosis distinct from the well-known insects with complete or holometabolous metamorphosis. Insects with incomplete metamorphosis are characterized by gradual body growth from hatching through the larval stage to the final sexually mature winged adult without major changes to the body plan. On the other hand, complete metamorphosis is characterized by the presence of the inactive and nonfeeding pupal stage between the highly appetitive larvae and the reproductive adults. Insects undergoing complete metamorphosis are prominent model species used in the laboratory, including *Drosophila melanogaster*. It is believed that the insects with incomplete metamorphosis are evolutionarily older than those with complete metamorphosis and that the ancestral species of insects were unable to undergo metamorphosis (Wipfler et al. 2019).

**Fig. 2:**
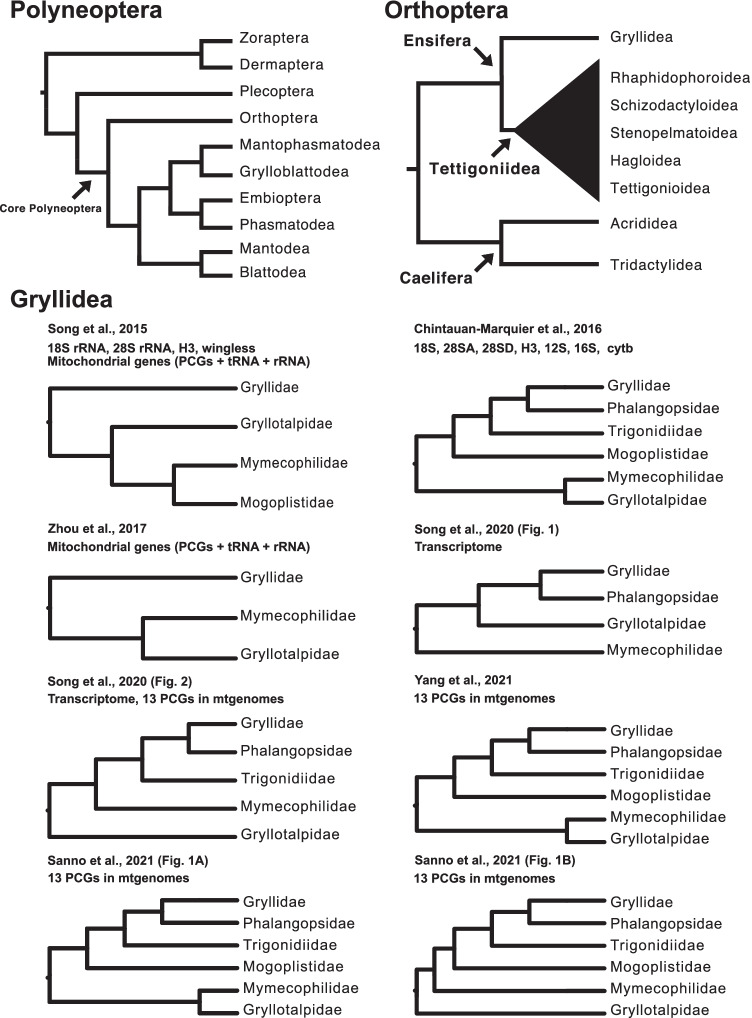
Phylogenetic trees of Polyneoptera, Orthoptera, and Gryllidea. The tree topology of Polyneoptera is based on Misof et al. 2014 and Wipfler et al. 2019, and of Orthoptera is on Zhou et al. 2017 and Song et al. 2020. Because there has been no or little consensus on the relationships within the Tettigoniidea, the lower taxonomic groups (Rhaphidophoroidea, Schizodactyloidea, Stenopelmatoidea, Hagloidea, and Tettigonioidea) are collapsed into one subtree. For Gryllidea, the inferred topological trees in recent studies have been shown

Phylogenetic relationships of the insects in Polyneoptera have long been controversial, which is due to a high degree of their morphological specializations (Whitfield and Kjer 2008). The phylogenetic study provides evidence for a monophyletic origin of Polyneoptera and its origin is estimated at 300 million years ago (Mya) or more (Misof et al. 2014). A recent study using protein-coding single-copy genes from public transcriptomic data has placed Dermaptera and Zoraptera as sister groups to the remaining orders; a group called “core Polyneoptera,” which comprises Orthoptera, Mantophasmatodea, Grylloblattodea, Embioptera, Phasmatodea, Mantodea, and Blattodea (Terry and Whiting 2005; Wipfler et al. 2019) (Fig. 2). Plecoptera is placed on a siter group of these “core Polyneoptera” groups, which diverged from an ancestor of Dermaptera and Zoraptera at approximately 270 Mya (Misof et al. 2014). Within the “core Polyneoptera” groups, Orthoptera is placed in the sister group that diverged from the remaining groups of “core Polyneoptera” at the earliest (Wipfler et al. 2019) (Fig. 2).

### Orthoptera

The orthopteran insects include grasshoppers, locusts, crickets, katydids, and their kins. Orthoptera is the most diverse and largest order in Polyneoptera, containing more than 28,900 extant species according to the Orthoptera Species File (Eades et al. 2014; Song et al. 2015) (http://orthoptera.speciesfile.org/). Three hundred million years of history has produced a stunning diversification of this order. Orthopteran insects are characterized by the presence of the cryptopleuron, a condition in which the pronotum covers a large part of the propleuron and by big jumping hind legs (Grimaldi and Engel 2005). This order is further divided into two suborders: Ensifera (e.g., crickets, katydids, and wetas) and Caelifera (e.g., grasshoppers and locusts). Ensifera is characterized by long, thread-like antennae, usually longer than the body, and thus are also known as “long-horned grasshoppers.” The female individual also features a sword‐like or needle‐like ovipositor. In contrast, Caelifera is characterized by “short-horned” antennae with less than 30 segments, whose female has an ovipositor with only two valvular pairs (Song 2018). One of the intriguing differences between Caelifera and Ensifera is the location of their auditory system; the insects belonging to Caelifera have their auditory organs in the first abdominal segment, whereas Ensifera have the organs in the tibiae of the prothoracic legs (Meier and Reichert 1990).

Within the Caelifera, there are two infraorders, which comprise more basal Acrididea and Tridaetylidea (Figs. 1 and 2). The former includes pygmy mole crickets (note that they do not belong to Ensifera, which “true crickets” belong to, although their common name contains “cricket”), while the latter includes grasshoppers and locusts. Ensifera has 16,000 or more species in approximately 2000 genera (Zhou et al. 2017) (Fig. 1). Most recent phylogenetic studies agree that Ensifera consists of two monophyletic infraorders: Gryllidea and Tettigoniidea (Song et al. 2015, 2020; Zhou et al. 2017) (Fig. 2). The former has more than 6000 valid species registered (Eades et al. 2014) (Fig. 1) and consists of Grylloidea (e.g., field crickets and sword-tailed crickets) and Gryllotalpoidea (e.g., mole crickets and ant-loving crickets). Tettigoniidea is the largest infraorder in Ensifera and includes Rhaphidophoroidea, Schizodactyloidea, Stenopelmatoidea, Hagloidea, and Tettigonioidea. Regarding the internal relationships of Tettigoniidea, there has not been a consensus among phylogenetic studies using different molecular markers (Eades et al. 2014; Song et al. 2015, 2020; Zhou et al. 2017) (Fig. 2).

### Gryllidea

The infraorder Gryllidea lists 6118 species according to the Orthoptera Species File (Eades et al. 2014) at the timing of writing the manuscript. The Gryllidea includes two superfamilies Grylloidea and Gryllotalpoidea. The latter consists of Gryllotalpidae (e.g., mole crickets) and Myrmecophilidae (e.g., ant-loving crickets). Within the Gryllidea, only 3–4% of species belong to the Gryllotalpidae. Grylloidea includes the family Gryllidae (true crickets), Mogoplistidae (scaly crickets), Trigonidiidae, Phalangopsidae, and other families. Gryllidae is the largest group in the Grylloidea, with over 3000 valid species registered.

The phylogenetic relationships within Gryllidea are not fully understood due to a long history of conflicting classification schemes (Fig. 2). Recent studies using the mitochondrial genome and a combination of nuclear and mitochondrial genes have shown that Grylloidea and Gryllotalpoidea are monophyletic sister groups (Song et al. 2015; Chintauan‐Marquier et al. 2016; Zhou et al. 2017; Yang et al. 2021; Sanno et al. 2021). However, other studies or approaches have either not confirmed this relationship or have produced conflicting phylogenetic relationships (Song et al. 2020; Sanno et al. 2021). To fully understand the phylogenetic relationship between Grylloidea and Gryllotalpoidea, we need genome sequences of a wide range of species included in this infraorder, which enable usage of single-copy gene sequences for building phylogenetic trees or of specific markers that accentuate the division of species.

## The most up-to-date status of cricket genomics and transcriptomics

Cricket genomes have been ever studied in various contexts, such as evolutionary biology, developmental and regenerative biology, and even entomophagy (Table 1). Out of the seven Orthoptera genomes registered in the National Center of Biotechnology Information (NCBI) Genome, the cricket genome that is currently sequenced consists of six species, namely *Laupala kohalensis* (Blankers et al. 2018a), *Teleogryllus oceanicus* (Pascoal et al. 2019), *T. occipitalis* (Kataoka et al. 2020), *Gryllus bimaculatus* (Ylla et al. 2021), *Acheta domesticus* (Gupta et al. 2020), and *Apteronemobius asahinai* (Satoh et al. 2021). The assembled size of current version of *A. domesticus* genomes registered in the NCBI (about 1.0 Gbp, GCA_014858955.1) is far less from that of about 2.0 Gbp predicted by photometric measurement (Lima-de-Faria et al. 1973; Gregory 2021). Gupta et al. sequenced a whole genome of *A. domesticus* not for its draft genome but for the development of microsatellite markers (Gupta et al. 2020). While genomes of *A. domesticus* were sequenced only on the short-read sequencer for de novo assembly, the genomes of the other five species have been sequenced on a combination of short-read and long-read based next-generation sequencers. The sizes of these five genomes range from 1.6 for *L. kohalensis* and *G. bimaculatus* to 2.0 Gbp for *T. occeanicus* and *T. occipitalis*. As of August 2021, despite the advancement of technologies to construct draft genomes at the chromosome level in animals and plants, there are still no chromosome-level genomes in the crickets. However, the studies on the cricket genomes that have been sequenced so far show a wide range of potential for basic and applied research. In this section, we outline the areas of research in which the genome and transcriptome information of cricket has been used.

**Table 1:** Recent studies on cricket genomics and transcriptomics

Research field	Species	Purpose	Reference
Evolutionary biology	*Gryllus firmus* and *G. pennsylvanicus*	Gene flow in hybrid zone	Maroja et al. 2009 Andrés et al. 2013 Larson et al. 2013 Larson et al. 2014 Maroja et al. 2015
	*Gryllus rubens* and *G. texensis*	Parapatric speciation	Gray et al., 2008 Blankers et al. 2018b Blankers et al. 2019b
	*Laupala* spp.	Coevolution of signal preference	Blankers et. 2018a Blankers et al. 2019a Xu and Shaw 2019 Xu and Shaw 2021 Ylla et al. 2021
	*Teleogryllus oceanicus*	Dosage compensation in sexual antagonism	Rayner et al. 2021
	*Teleogryllus oceanicus*	Early stage of adaptive and parallel evolution (flatwing)	Pascoal et al. 2014 Pascoal et al. 2018 Pascoal et al. 2019 Zhang et al. 2021
Phenotypic plasticity	*Teleogryllus oceanicus* and *T. commodus*	Evolution in X chromosome	Moran et al. 2018
	*Gryllus firmus*	Wing polymorphism	Nanoth Vellichirammal et al. 2014 Zera et al. 2018
	*Gryllus pennsylvanicus*	Cold tolerance	Des Marteaux et al. 2017
	*Gryllus veletis*	Cold tolerance	Toxopeus et al. 2019a
Compensatory plasticity	*Gryllus bimaculatus*	Structural neuronal changes in the auditory system	Fisher et al. 2018
	*Gryllus bimaculatus*	Synaptic strength change in the cercal escape circuitry	Prasad et al. 2021
Developmental and regenerative biology	*Gryllus bimaculatus* and *G. rubens*	Leg regeneration	Bando et al. 2013
	*Gryllus bimaculatus* and *G. rubens*	Stage-specific transcriptome	Zeng et al. 2013 Berdan et al. 2016 Kono et al. 2021
Entomophagy	*Acheta domesticus*	Domestication	Oppert et al. 2020
	*Gryllus bimaculatus*	Domestication	Ylla et al. 2021
	*Teleogryllus occipitalis*	Domestication	Kataoka et al. 2020
Others	*Apteronemobius asahinai*	Circatidal rhythm	Satoh et al. 2019 Satoh et al. 2021
	*Gryllus bimaculatus*	Codon usage frequency and expression level	Whittle et al. 2021b
	*Gryllus bimaculatus*	DNA methylation	Ylla et al. 2021
	*Gryllus bimaculatus*	Sex-biased gene expression regulation	Whittle et al. 2021a

### Evolutionary biology

Some of the cricket species have been studied for understanding the genomic structures underlying speciation. The Hawaiian swordtail cricket genus *Laupala* is one of the groups of crickets that has been most intensively studied for genomic architectures of reproductive isolation and speciation. The *Laupala* cricket is rapidly radiating and thus endemic to each of the single islands in the archipelago, resulting in 38 species. The male *Laupala*’s mating song has a species-specific pulse rate that attracts females. In contrast, female *Laupala* shows an acoustic preference for the pulse rate of the same species. The rapid divergence and coevolution of the mating songs and conspecific acoustic preferences has been thought to be a driving force of sexual selection, thus making this species a powerful system for dissecting the basis for parallel and convergent evolution. *L. kohalensis* is the first species of cricket for which a complete genome sequence has been published (Blankers et al. 2018a). The draft genome of *L. kohalensis* was used to examine local variation in recombination rates across the genome and in relation to the location of the song and preference quantitative trait locus (QTL). The results showed that, contrary to the hypothesis that the suppression of interspecific recombination promotes the coevolution of song and preference, the genomic regions where male song and female preference QTL coexist did not have particularly low recombination rates (Blankers et al. 2018a). Further study has revealed that the repeated song-preference coevolutions in independent *Laupala* species share the same QTLs (Blankers et al. 2019a). Furthermore, the QTLs associated with male mating songs and female preference have been shown to be closely coupled, suggesting genetic coupling, and include genes underlying variation in song and preference divergence, such as genes involved in the neuronal formation, rhythmic muscle activities, and signal processing in auditory systems (Xu and Shaw, 2019; Xu and Shaw, 2021). Recent analysis on gene family expansion/contraction has revealed the cricket-specific pickpocket gene family, which encodes ion channels and is involved in locomotion, neural modulation, and muscle development (Younger et al. 2013; Hill et al. 2017; Ylla et al. 2021). *L. kohalensis* has been shown to contain pickpocket genes in a genomic region associated with changes in song rhythm, possibly suggesting that song production in crickets is likely to be regulated by ion channels (Ylla et al. 2021).

*Teleogryllus oceanicus* is another model for evolutionary genomics. *T. oceanicus* also habitats in the Hawaiian Islands, and the male individuals adaptively lose their songs to protect them from a lethal, eavesdropping parasitoid fly *Ormia ochracea* (Zuk et al. 2006). The female fly identifies the location of male crickets by hearing their chirp and lays its eggs inside the crickets. The fly larvae grow inside the cricket and when they emerge a week or later, they kill their host. The silent population of the crickets was first observed in 2003 on Kauai and then rapidly spread in fewer than 20 generations (with three to four generations per year) to near-fixation, which thus reflects the early stage of adaptive evolution. This genetic variant called “flatwing” was linked with the X chromosome (Tinghitella 2008). Interestingly, flatwing has also appeared on the different Hawaiian Islands, and these similar phenotypes have been associated with morphometrically distinct wings and shown to be caused by independent mutational events but not by genetic introgression between geographically different populations, as revealed by restriction associated DNA tag sequencing (RAD-seq) and genome-wide association study (GWAS) supported by high-coverage resequencing data (Pascoal et al. 2014; Zhang et al. 2021). Nevertheless, it has been shown that the populations on different islands have experienced recent and ongoing human activity-related gene flow, which theoretically counteracts the generation and maintenance of parallel adaptations (Zhang et al. 2021). The whole genome of this species has indeed revealed a flatwing QTL covering a large region of the X chromosome (Pascoal et al. 2019). This study also found that the flatwing male expresses different profiles of chemical pheromones, which is similar to the female ones, and this change is genetically associated with the X chromosome loci responsible for the flatwing phenotype, suggesting abrupt adaptation through pleiotropy or genomic hitchhiking (Pascoal et al. 2019). In other recent studies, genomic and transcriptomic information in *T. oceanicus* has been used to support the theory that phenotypic plasticity drives adaptive evolution (Pascoal et al. 2018) and to study the role of dosage compensation in sexual antagonism (Rayner et al. 2021).

Cricket has also been used as a model to study the genetic basis of parapatric or sympatric speciation that occurs within a continent, unlike allopatric speciation between the islands as described above. For example, two sexually isolated species, *Gryllus* *rubens* and *G. texensis*, are distributed in adjacent areas of the Eastern and Southern USA, forming a hybrid zone. They diverged 500,000 years ago followed by a long period of bidirectional gene flow that lasted until 18,000 years ago, as revealed by sequence data of gene for mitochondrial cytochrome oxidase I and transcriptome-based SNP data (Gray et al. 2008; Blankers et al. 2018b). The parapatric origin of *G.* *rubens* has been demonstrated, and a mechanism for the coevolution of mating song and preference has been proposed (Blankers et al. 2018b, 2019b). Like this, other studies have utilized the field crickets *G. firmus* and *G. pennsylvanicus*, which interact and form a hybrid zone in the Eastern USA. They diverged more recently, 200,000 years ago, and there is an ongoing gene flow despite prezygotic barriers (Maroja et al. 2009; Larson et al. 2013). Transcriptomic scan from individuals in a hybrid zone reveals candidate prezygotic barrier genes with remarkably reduced introgression including ones encoding cytoskeletal proteins, which are involved in critical steps in fertilization (Andrés et al. 2013; Larson et al. 2013). The loci involved in speciation were speculated to be located on the X chromosome, which has been confirmed by a backcross experiment to construct a genetic linkage map for these species (Larson et al. 2014; Maroja et al. 2015). Another example includes a pair of *T. oceanicus* *and T. commodus*, which are close species on the eastern coast in Australia. RAD-seq-based comparative study about genetic variation at X and autosomal loci within and between the species poses a challenge to the theory that sex chromosomes are predicted to exhibit reduced diversity and greater divergence between species and populations compared to autosomes (Moran et al. 2018).

### Phenotypic plasticity

Wing pleomorphism is a phylogenetically widespread and ecologically important feature of insects. The sand cricket *Gryllus firmus* exhibits a well-known wing polymorphism, consisting of a long-winged morph with functional flight muscles that is capable of flight, and two flightless morphs. The two flightless morphs differ in the timing of becoming flightless, but both morphs show more pronounced fertility than the flight-capable morphs by the end of the first week of adult life. Thus, this species is an orthopteran model for studying a life-history trade-off between reproduction and flight capability in females. This polymorphism is polygenically and environmentally controlled, conferring morph-specific biochemical, physiological, and behavioral differences such as hormone level and energy consumption (Zera 2009). To better understand the molecular basis of these morph-specific adaptations, a de novo transcriptome analysis on different morphs has been conducted (Nanoth Vellichirammal et al. 2014). This research has identified genes involved in this polymorphism in flight muscle and fat bodies, including genes related to respiration, proteolysis, triglyceride biosynthesis, lipid transport, immune function, and reproduction (Nanoth Vellichirammal et al. 2014). Interestingly, RNA-seq analysis also shows that flight-capable morphs have a significantly higher number of genes that exhibit diurnal changes in gene expression compared to flightless morphs (Zera et al. 2018). This model can be utilized as a useful empirical model to study circadian aspects of life-history adaptation.

Moreover, the transcriptomics of some species has contributed to the understanding of the mechanisms of cold tolerance in insects. Crickets originate from the tropics, but many species are also found in temperate zones and have their overwintering strategies. The fall field cricket, *G. pennsylvanicus*, is univoltine and overwinters in diapause in the soil as an egg. Although rapid cold hardening induces loss of homeostasis of ion and water and eventually death in *G. pennsylvanicus*, this species exhibits plasticity under cold acclimation (Coello Alvarado et al., 2015). Tissue-specific transcriptomic analysis shows that this species acquires cold tolerance by regulating the expression of genes encoding proteins involved in the transport of substances between hemolymph and gut (Des Marteaux et al. 2017). The spring field cricket, *G. veletis*, is also a promising model for mechanistic studies of insect freeze tolerance. A laboratory model which mimics autumn-like conditions in nature induces freeze tolerance in the late instar juveniles, which is accompanied by gene expression regulation that may contribute to metabolic suppression as well as elevated membrane and cytoskeletal remodeling, cryoprotectant transporters, cytoprotective proteins, and antioxidants (Toxopeus et al. 2019a, b).

### Compensatory plasticity

The nervous system has the potential to adapt to the loss of one modality by making plastic changes to its structure, connectivity, function, and neural interactions. The auditory system of cricket has the capacity to respond to deafferentation even as an adult, through compensatory dendritic growth and subsequent synapse formation (Horch et al. 2011). Auditory interneurons, such as ascending neuron 2 (AN-2) in *G. bimaculatus*, normally have dendrites that extend to, but not over, the midline of the prothoracic ganglion. However, after unilateral removal of the ear, the dendrites of the ipsilateral AN-2 are dramatically altered, with the medial dendrite extending beyond the midline to form a compensatory synapse with auditory afferents from the contralateral ear. De novo transcriptome analysis on cricket prothoracic ganglia has identified several well-conserved guidance molecules in neuronal wirings such as slit, netrin, ephrin, and semaphorin (Fisher et al. 2018). In addition to compensatory plasticity in the auditory system, crickets are also known to have the compensatory response of the escape behavior mediated by cerci, two antenna-like appendages located on the posterior end of the abdomen. Each cercus detects and integrates directional information on airflow originating from a predator with mechanoreceptor hairs, triggering an appropriate escape response. Activation of the hairs on one side of the cercus stimulates the ipsilateral ascending giant interneurons, while the contralateral giant interneurons respond only slightly. However, when the ipsilateral cercus is removed, the giant interneurons from the remaining contralateral cercus receive a stronger than normal excitatory signal. Unlike the auditory system, the compensatory plasticity in the cercal escape circuitry does not accompany the structural changes in dendrites and instead relies on shifts in excitatory and inhibitory synaptic strength. A terminal ganglion transcriptome in crickets after unilateral cercal ablation has revealed enrichment of upregulation of genes involved in the ubiquitin-proteosome protein degradation system, chromatin-mediated transcriptional pathways, and GTPase-related signaling system (Prasad et al. 2021).

### Developmental and regenerative biology

The field cricket has been used as a model for understanding developmental mechanisms in hemimetabolous insects (Donoughe and Extavour 2016). Although there are many transcripts of unknown function, the transcriptomic data on oogenesis, embryonic development, and instar development are available for two field cricket species, *G. bimaculatus* and *G.* *rubens* (Zeng et al. 2013; Berdan et al. 2016; Kono et al. 2021). *G. bimaculatus* is emerging as a model for regeneration research. In this cricket, distal parts of the amputated leg are regenerated in response to injury. A key cell population called blastema, which has the pluripotent proliferative capacity, proliferates and differentiates to restore the lost parts of tissues. RNA-seq-based global gene expression profile during the early regeneration process has identified the JAK/STAT signaling pathway as a key signaling pathway in blastema formation (Bando et al. 2013).

### Entomophagy

Cricket is emerging as a future source of animal protein for human consumption and for vertebrate livestock. The crops and livestock that we humans eat today are the results of a long process of selection, which has conferred ideal traits, such as size, growth rate, stress resistance, and organoleptic properties, on the livestock. As will be discussed in the later sections, genome and transcriptome information on crickets will contribute to the domestication of crickets and to the food production supported by insects. The genome of *T. occipitalis* was sequenced by the authors, explicitly intending to be applied for the first time to an alternative animal protein for human food and animal feed (Kataoka et al. 2020). *T. occipitalis* is mentioned in the Food and Agriculture Organization of the United Nations (FAO) report and is one of the edible species reared in Asian countries (van Huis et al. 2013). This species inhabits the southwestern islands in Japan, China, and Southeast Asian countries. The two-spotted cricket *Gryllus bimaculatus* and house cricket *Acheta domesticus* are probably the most widely distributed food and feed crickets in the world today. As of November 2021, genome information of *G. bimaculatus* and transcriptome information of *A. domesticus* are available (Oppert et al. 2020; Ylla et al. 2021). Crickets are rich in nutrients, but this value could be further enhanced by improving their vitamin content and production of Omega-3 fatty acids. Genetic information related to immunity could also help to prevent infectious diseases in crickets (Hussain et al. 2020).

### Others

Field crickets are also used in studies of gene expression regulation in hemimetabolous insects. In the recent study of *G. bimaculatus* genome, the pattern of DNA methylation common to holometabolous and hemimetabolous insects is mainly reported (Ylla et al. 2021). DNA methylation is an important DNA modification exclusively found in cytosine residues in eukaryotes, which is responsible for genome integrity and regulation of gene expression. Contrary to the mammalian pattern of DNA methylations, in which they occur throughout the genome except for CpG islands near promoters of genes (Suzuki and Bird 2008), DNA methylations in insect genomes frequently occur in the gene bodies (Bonasio et al. 2012; Feng et al. 2010), regulating behavioral plasticity and social behavior. Studies on CpG depletion, considered a reliable indicator of DNA methylation, have revealed that DNA methylated genes tend to perform housekeeping functions across insects including holometabolous and hemimetabolous ones (Ylla et al. 2021). In addition to the epigenomics described above, the genome and transcriptome in crickets have been used for studying the sex-biased gene expression regulation, which is linked to protein sequence evolution (Whittle et al. 2021a) and the relationship between codon usage frequency and expression level in different tissues and sexes (Whittle et al. 2021b).

Another line of studies on cricket genome and transcriptome involves biological rhythm. The mangrove cricket *Apteronemobius asahinai*, which is endemic to mangrove forest floors, shows an endogenous activity rhythm with the cycle of approximately 12.4 h formed by tidal flooding and ebbing (circatidal rhythm). RNA-seq analysis has detected a few hundreds of significant circatidal cycling transcripts, including genes encoding antioxidants and detoxification enzymes (Satoh and Terai, 2019). The draft genome of this species has been recently sequenced by the hybrid Illumina/Nanopore sequencing technologies, which will accelerate molecular research in the studies on circatidal rhythm (Satoh et al. 2021).

## Genomic resources of polyneopteran insects

Since the publication of the genome sequence of *D. melanogaster* in 2,000 (Adams et al. 2000), insect genomics has revolved largely around holometabolous insects. Hemimetabolous insects are of great importance for the study of external morphology, social behavior, lifestyle, and early evolution of winged insects, yet their study is still far behind that of holometabolous insects. In terms of genomic information, the number of species of hemimetabolous insects with genomic data registered in the NCBI is 101 as of August 2021, which is much less than 1305 for holometabolous insects (Table 2). Most of the genomic data for hemimetabolous insects is so far from Hemiptera, as represented by pea aphid *Acyrthosiphon pisum*. The genome of this species has been sequenced for the first time in hemimetabolous insects to study agricultural pests and symbiosis (The International Aphid Genomics Consortium 2010). The number of genomic resources of polyneopteran insects is now 31, among which Orthoptera is just seven (Table 2). Previous studies have been biased toward holometabolic insects and the limited information on the genome of Polyneoptera has undoubtedly hampered elucidation of the evolutionary history of the insect as a whole. However, recent advancements in long-read sequencing technologies and computational analysis have opened the possibility of large-scale analysis of genomes diversified from Polyneoptera (Hotaling et al. 2021). Here, we focus on the Polyneopteran genomes and analyze the characteristics of their genomes especially their genome size, chromosome number, and repetitive regions.

**Table 2:** Registration number of genomes of holometabola and hemimetabola in the NCBI

Order	Taxonomy ID﻿	Number of genomes in the NCBI (August 2021)
*Holometabola* (1305)		
Lepidoptera	7088	736
Diptera	7147	272
Hymenoptera	7399	210
Coleptera	7041	75
Trichoptera	30263	8
Megaloptera	50553	1
Neuroptera	7516	1
Siphonaptera	7509	1
Strepsiptera	30261	1
Mecoptera	27420	0
Raphidioptera	50482	0
*Hemimetabola–Polyneoptera* (31)		
Phasmatodea	7020	13
Orthoptera	6993	7
Blattodea	85823	6
Plecptera	50622	4
Dermaptera	27434	1
Embioptera	50657	0
Grylloblattodea	58557	0
Mantodea	7504	0
Mantophasmatodea	192413	0
Zoraptera	30265	0
*Hemimetabola–non-Polyneoptera* (70)		
Hemiptera	7524	60
Ephemeroptera	30073	4
Odonata	6961	3
Thysanoptera	30262	3
Insecta†	50557	1429

^†^Insecta represents the orders above plus the remaining orders: Protura, Collembola, Diplura, Archaegnatha, Zygentoma, and Psocodea

### Genome size of polyneopteran insects

**Fig. 3:**
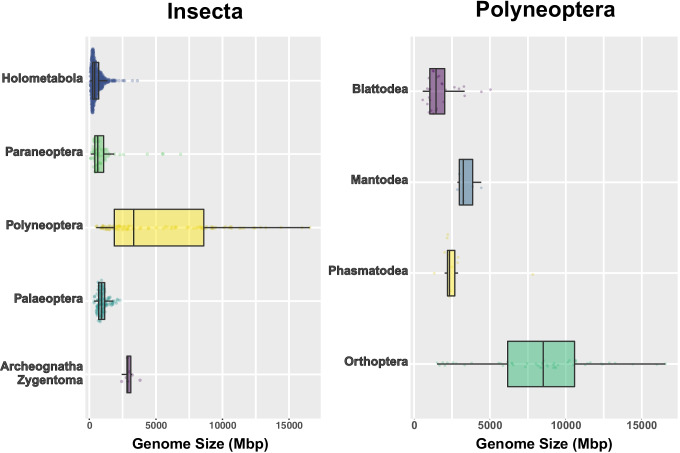
Genome size variation in Insecta and Polyneoptera. The data on the genome size were collected from the Animal Genome Size Database (https://www.genomesize.com) (Gregory 2021)

The genome size, or *C* value, is defined as the amount of haploid DNA per cell (Greilhuber et al. 2005). It is typically expressed in picograms (pg) or basepairs (bp), and their relation was established as 1 pg = 978 Mbp DNA (Dolezel et al. 2003). One of the reasons for the paucity of genomic data on Polyneoptera is probably attributed to its large genome size, making it challenging to sequence their entire genomes. Most holometabolan species have a relatively small genome among insects, ranging from 68.5 Mbp to 3.61 Gbp (Fig. 3), as exemplified by about 140 Mbp of a model organism *D. melanogaster* (Adams et al. 2000). Conversely, hemimetabolous species, particularly polyneopteran species, possess a wider range of genome size (Fig. 3), which ranges from 518 Mbp to 16.6 Gbp. The species with the largest genome size in insects in the Animal Genome Size database (Gregory, 2021) is *Podisma pedestris* (Orthoptera; Acrididae) with 16.93 pg (16.56 Gbp), while the smallest genome size is 0.07 pg (68.46 Mbp) in *Clunio tsushimensis* (Diptera; Chironomidae). Given that the number of genes carried by species in Polyneoptera is essentially constant, comparison of their genomes will be of great use in studying the evolution of genome size. Analysis of the distribution of genome sizes in Polyneoptera including Blattodea, Mantodea, Phasmatodea, and Orthoptera, which have sufficient sample sizes, shows that the genome size of Orthoptera is remarkably variable (Fig. 3). This suggests that the genome size diversity of Polyneoptera should be largely due to that of Orthoptera. The genome size of Gryllidea ranges from 1.55 Gbp in *Hadenoecus subterraneus* to 2.25 Gbp in *Gryllus pennsylvanicus*, which is a relatively small subset of Orthoptera. Indeed, several lines of comprehensive studies also have suggested that genome sizes of insects in Gryllidea are in the minimum range for orthopterans (Hanrahan and Johnston 2011; Yuan et al. 2021). Considering that the median genome size of insects is 518.3 Mbp, the genome size of Gryllidea is quite large. Note that among the datasets of genome size of about 1160 insects, only about 50 species of Orthoptera are described, and of these, 40 species belong to the family Acrididae (Caelifera). For Gryllidea, there are data from only six species. We believe that unbiased datasets will allow for more sound insights in the future.

Genome size has a significant impact on a variety of fitness-associated parameters, including growth, metabolism, life-history traits, and body size (Dufresne and Jeffery 2011; Alfsnes et al. 2017). In general, as genome size increases, cell size increases, which is a common tendency found in almost all organisms (Cavalier-Smith 1985; Gregory 2002). An increase in cell size results in an increase in the duration of cell division (Bennett 1977). In many organisms, including insects, it is known that the longer the duration of cell division, the slower the growth rate (the longer time from hatching to adulthood in insects) (Alfsnes et al. 2017). Hence, genome size is negatively correlated with the developmental rate (positively correlated with the developmental duration). For most animals, the duration of development influences adult body size. To increase the size of the body, it is necessary to either increase the development time or increase the growth rate (Roff 2000). Experimental data using reverse genetics in a cricket *Gryllus bimaculatus* provided evidence that the body size of adults increases with the duration of their developmental period (Ishimaru et al. 2016). Therefore, the great diversity of genome size in Orthoptera might be linked to the varying sizes of their body. Interestingly, Orthoptera, along with Odonata (e.g., dragonflies) and Psocodea (e.g., lice), are the group of insects with the largest deviation in their body sizes (Rainford et al. 2016). For instance, while there are ant-loving crickets (Myrmecophilidae), which have the smallest body size in all Orthopteran species, with adults measuring only 2–3 mm, a giant weta *Deinacrida heteracantha* (Ensifera; Tettigoniidea) from New Zealand is the heaviest species in Orthoptera with females weighing up to 71 g (Williams 2001) (Supplementary Table 1). Assuming that the weight of Myrmecophilidae ant-loving crickets is 2 mg, the difference in weight between the smallest and the largest Orthoptera species is 35,000-fold (Whitman 2008). Since body size and mass strongly correlate with fitness (Whitman 2008), the flourishing of species in Orthoptera with diverse ecological niches (> 28,900 described species) possibly reflects the tunability of their genome size. Consistently, in flatworms and copepods, a strong association between genome size and body size has been reported (Gregory et al. 2000). However, a study that examined genome size and body size in Tettigoniidae species did not find a significant positive correlation between them, but it may need to be tested more rigorously with a larger sample size (Yuan et al. 2021). It would be interesting to investigate the mechanisms that allow for variation in genome size.

### Chromosome number of polyneopteran genomes

One of the reasons that the genome sizes dramatically change may be attributed to chromosome-level structural changes. Chromosome number is a fundamental characteristic of a genome. Changes in the number of chromosomes are caused by the division or fusion of chromosomes (de Vos et al. 2020), which ultimately promotes speciation. Decrease in the number of chromosomes can be caused by the loss of short nonessential DNA after the division of acrocentric chromosomes at centromeres (termed as “Robertsonian translocations”) or by the fusion of telomeres of two chromosomes followed by the loss of centromeres on one of the chromosomes (Gordon et al. 2011). Conversely, an increase in the number of chromosomes can be caused by chromosomes dividing at or near the centromere (Moretti and Sabato 1984) or by a duplication of entire chromosomes. Although unusual in most groups of animals, whole-genome duplication can double the number of chromosomes (Beçak et al. 1970; Doyle et al. 2008). Chromosome number has a broad impact on many aspects of the biological system such as gene transcription (Sun et al. 2013), recombination rates (Dumont 2017), and sex chromosome evolution (Blackmon et al. 2017). For the linkage between chromosome number and sex determination, it has been found that species with many small chromosomes in beetles Coleoptera tends to shift from the XY system to the XO system rather than those with a small number of large chromosomes, which is thought to be due to the event of the loss of a small, nonessential Y chromosome (Blackmon and Demuth, 2015).

**Fig. 4:**
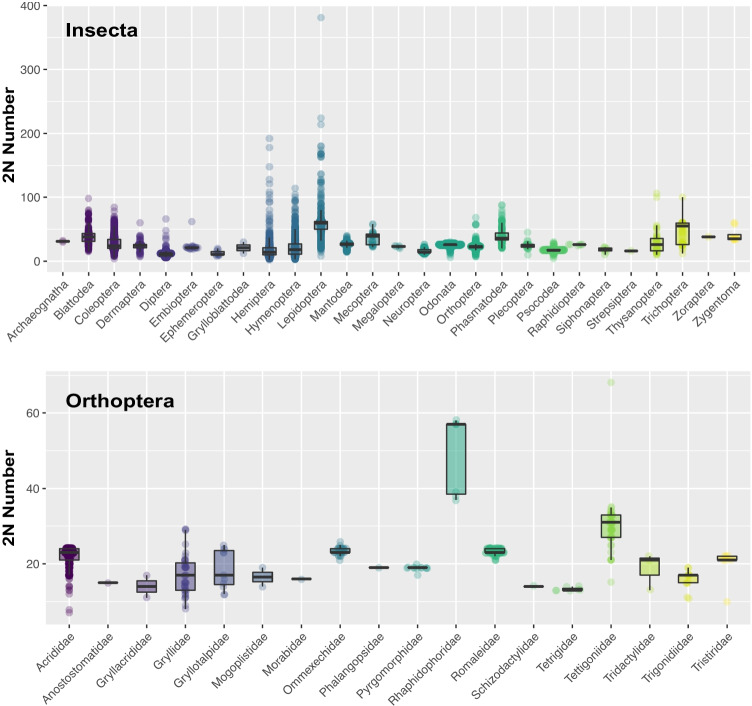
Diploid chromosome number of Insecta and Orthoptera. The data on the chromosome number were collected from the Animal Chromosome Count database (https://cromanpa94.github.io/ACC/) (Román-Palacios et al. 2021)

Insects vary enormously in chromosome number. Among insects, Lepidoptera (e.g., butterflies and moths) represents large intraspecific and interspecific variations in the number of chromosomal diploids with about *2n* = 58 on average (Lukhtanov et al. 2011) (Fig. 4, Supplementary Table 2), according to the Animal Chromosome Count database (https://cromanpa94.github.io/ACC/) (Román-Palacios et al. 2021). Although most groups of Lepidoptera, just like other animals and plants, have a relatively stable chromosome number, there are several families, for example, Lycaenidae (Talavera et al. 2013) and Papilionidae (Emmel et al. 1995), that have an unusually large number of chromosomes. For example, the blue butterfly species *Polyommatus atlanticus* (Lycaenidae) has the largest number of chromosomes in the non-polyploid eukaryotic organisms, with at least *n* = 224–226 countable chromosome bodies (Lukhtanov 2015). In contrast, the Diptera, which has long been analyzed as a model organism among insects, has about *2n* = 11 on average. The diversity of chromosome numbers across taxa can be explained by the difference in the rate of chromosomal mutations such as fusion, fission, and translocation. Indeed, genome rearrangement rates are significantly higher in Lepidoptera than in Diptera by an order of magnitude (d’Alencon et al. 2010). The remarkable diversity in chromosome number in Lepidoptera was long thought to be due to chromosomal holocentricity (multiple kinetochores along their length rather than the single centromere), which enables the successful inheritance of novel fission or fusion fragments (Lukhtanov et al. 2018; Hill et al. 2019). However, the recent analysis suggested that chromosome number is independent of whether they have holocentric or monocentric chromosomes in any taxa of insects (Ruckman et al. 2020).

In polyneopteran insects, the number of chromosomes varies depending on the order. According to the analysis using the datasets deposited on the Animal Chromosome Count database, Phasmatodea and Blattodea have many species with chromosome diploid numbers greater than 2*n* = 50, contributing to the extreme diversity of karyotypes in this order (Fig. 4, Supplementary Table 2). Phasmatodea and Blattodea have an average chromosome number of 2*n* = 40, which is clearly larger than the chromosome number of the remaining orders in Polyneoptera, which has an average of 2*n* = 25, suggesting that they have a higher rate of chromosomal fission events than the other polyneopteran groups (Sylvester et al. 2020) (Fig. 4, Supplementary Table 2). Orthopteran diploid chromosome number is 2*n* = 22.5 on average and is less variable. Thus, Orthoptera is likely the order with the largest genome size relative to its small number of chromosomes in the polyneopteran insects (Sylvester et al. 2020). Within Orthoptera, Rhaphidophoridae exhibits a relatively high number of chromosome numbers, reaching a maximum of 2*n* = 58 in *Diestrammena japanica* (Makino 1931) (Fig. 4). The chromosome number of Gryllidae shows a relatively wider variety rather than the other orthopteran lineages, ranging from 2*n* = 8 in *Eneoptera surinamensis* to 2*n* = 29 in *Gryllus assimilis*, *G. bimaculatus*, and *G. campestris* (Fig. 4). One possible explanation for this variety of chromosome numbers can be linked to ancient polyploidy in the ancestors of orthopteran sublineages; there is, however, no evidence of ancient genome duplications in the genomes of hexapods except Lepidoptera, Trichoptera, and Odonata (Li et al. 2018). Thus, it is reasonable to assume that the large variation in genome size in Orthoptera is not due to variation at the chromosome level but to some other potential factors. The diversity in chromosome number in Orthoptera, although not as much as in Lepidoptera, is expected to have been partly caused by chromosome fusion and division, which requires further investigation in the future.

### Repeat sequences in polyneopteran genomes

Another underlying potential mechanism of diverse genome size in Orthoptera is involved in repetitive regions, in particular transposable elements (TEs), in their genomes. TEs are DNA sequences that can jump in host genomes and are widespread in eukaryotic organisms and are generally deleterious since they give rise to mutations that lead to sterility and malformations (Gilbert et al. 2021). TEs fall into two major classes (Finnegan 1989). Class I elements, or retrotransposons, replicate and transpose via an RNA intermediate, which is often termed as the “copy and paste” mechanism of transposition. Retrotransposons include long and short interspersed elements (LINEs, SINEs) and long terminal repeat (LTR) retrotransposons. In contrast, class II elements, also known as DNA transposons, use DNA intermediates in their “cut and paste” mechanism. DNA transposons have structural features that are unique to their superfamily. For example, *hAT* (*hobo*-*Ac*-*Tam3*) superfamily, which includes *hobo* elements in *D. melanogaster*, *Activator* elements in maize and *Tam3* elements in *Antirrhinum majus*, is characterized by DNA consensus sequence corresponding to a domain of approximately 50 amino acids located at the C-terminus. (Rubin et al. 2001). Furthermore, the *P* element in *D. melanogaster* is one of the most famous DNA transposons, which causes hybrid dysgenesis, a phenomenon whereby F1 hybrids between males harboring *P* elements and females lacking them shows several defects such as gonadal atrophy and sterility (Bucheton et al. 1984). Although TEs are viewed as “junk DNA” as a nonessential region of the genome in animals and plants, recent accumulating studies have updated this old view and revealed that they involve genome rearrangement, transcriptional regulation, and even immunity (Bourque et al. 2018). For example, TEs can disrupt coding sequences or gene regulation by transposing host genes or regulatory sequences. In other cases, chromosomal rearrangements (e.g., deletions, duplications, inversions, and translocations) can be induced by ectopic (nonhomologous) recombination. In insects, TEs are involved in the acquisition of insecticide resistance (Chen and Li 2007; Itokawa et al. 2010) and the evolution of climate adaptation (González et al. 2010; Kim et al. 2014).

Comparative analyses of TE composition in insects have been conducted on several model organisms. For example, a comparison of 26 *Drosophila* species genomes has detected a strong phylogenetic signal on the evolution of both genome size and TE content (Sessegolo et al. 2016). Despite the ever-growing number of insect genomes sequenced to date, comprehensive study on the diversity and evolution of insect TEs remains surprisingly poor. In recent studies, in which a broad range of insect groups was used, TE contents have been found to vary greatly among the groups and even between species belonging to the same orders (Petersen et al. 2019; Wu and Lu 2019). These studies have also confirmed that the genome size was positively correlated with TE content and diversity (Petersen et al. 2019; Wu and Lu 2019). The migratory locust *Locusta migratoria*, which belongs to Orthoptera and has the largest genome size of any insect sequenced to date (Wang et al. 2014), has been found to have 61 different TE superfamilies, which is the largest TE diversity examined, with the mean of 54.8 TE superfamilies in insects (Petersen et al. 2019). The TE landscape in Polyneoptera is dominated by LINEs (Petersen et al. 2019), although many species-specific TEs that are less represented in the database used in the study (Repbase) (Bao et al. 2015) are not well-covered.

**Fig. 5:**
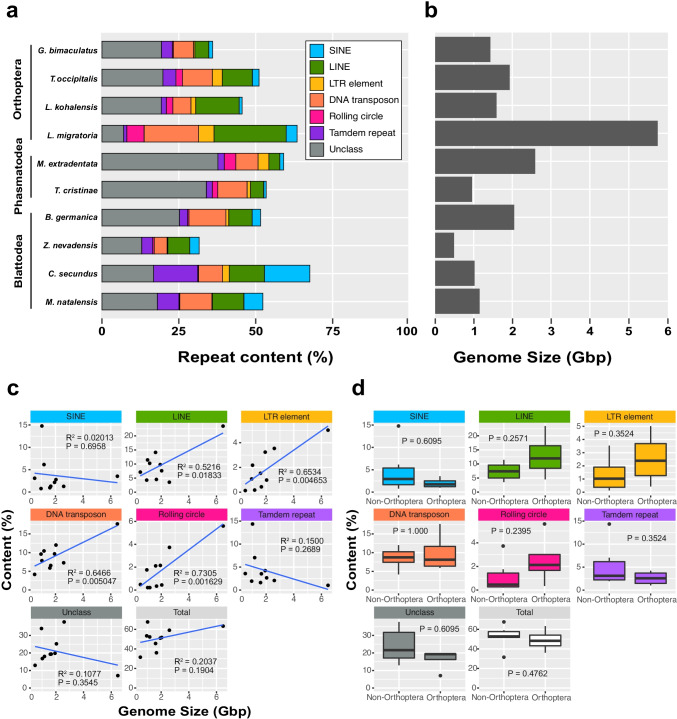
Repeat content and genome size in Polyneoptera. **a** Repetitive contents of the polynepteran genomes were analyzed by RepeatMasker (Smit et al. 2015) using Repbase (edition 20170127) (Bao et al. 2015) and Dfam database (Storer et al. 2021). The sources of the genomes used in the analysis are as follows: *Gryllus bimaculatus*, GCA_017312745.1; *Teleogryllus occipitalis*, GCA_011170035.1; *Laupala kohalensis*, GCA_002313205.1; *Locusta migratoria*, GCA_000516895.1; *Medauroidea extradentata*, GCA_003012365.1; *Timema cristinae*, GCA_002926335.1; *Blattella germanica*, GCA_003018175.1; *Zootermopsis nevadensis*, GCA_000696155.1; *Cryptotermes secundus*, GCA_002891405.2; *Macrotermes natalensis*, GigaDB (http://gigadb.org/dataset/). **b** The genome sizes of the indicated species belonging to Polyneoptera in **a**. **c** Correlation between the genome size and each type of repeat sequence. The species used in the analysis are the same as those used in **a** and **b**. **d** Comparison of the percentage of each repeat sequence in Orthoptera and non-Orthoptera

We used the recently published genome information of polyneopteran insects to reanalyze the diversity of repetitive sequences, including TEs (Fig. 5, Supplementary Table 3). The relative repeat content of genomes from ten species covering three orders (Orthoptera, Phasmatodea, and Blattodea), and their genome sizes were shown in Figs. 5a and b (Supplementary Table 4). In most species, “unclassified” elements, which need further characterization, represent the largest fraction. We further examined the relative contribution of the major repetitive elements (i.e., SINE, LINE, LTR elements, DNA transposons, rolling circles, and tandem repeats) to the genome sizes in Polyneoptera and found that LINE, LTR elements, DNA transposon, and rolling circles may contribute to the expansion of genome size in Polyneoptera (Fig. 5c). We also observed no correlation between the genome size and total repeat contents, which may be due to exceptionally large repeat content for the genome sizes in *C. secundus* and *M. natalensis.* Furthermore, we compared the relative amount of each type of repetitive region between Orthoptera and non-Orthoptera, yet found no significant differences in any of the repeat regions (Fig. 5d). These results suggest that the repetitive content has little impact on the genome size expansion in Orthoptera, but it should be noted that sample sizes are small (especially for grasshoppers and locusts with extremely large genomes). In addition, it should be noted that the draft genomes studied here are sequenced by different sequencing technologies and assembled by different methods (Supplementary Table 3), which leaves the possibility that not all repeat sequences in the polyneopteran draft genomes have been correctly reconstructed. Overall, the several types of repetitive contents may contribute to the genome size expansion in Polyneoptera, although investigating the contribution of repeat sequences to genome size in Orthoptera requires more sample size and high-quality genome sequences.

Repetitive sequences in the genome can cause chromosome breaks and structural rearrangements that alter the number of chromosomes (Carbone et al. 2014). While small genomes have a low copy number of TEs that are generally constrained, large genomes have greatly varying numbers of TE copies (Kidwell, 2002). However, it has been suggested that there is no correlation between chromosome number and genome size in Polyneoptera (Sylvester et al. 2020). Indeed, the genome of Orthoptera, which contains a large number of TEs, has a huge genome size, but the diploid chromosome number is relatively stable within the order, averaging around 22.5 (Fig. 4). Thus, it is likely that the large size of the orthopteran genome is not related to chromosomal rearrangements that depend on the abundance of repetitive structures.

### Biological impact of genome size diversity in Orthoptera

Finally, we discuss how the diversity of orthopteran genome size can be involved in biology and evolution. A study that brings a phylogenetic view to the comparison of genome sizes in orthopteran insects suggests that the genome size of Tettigoniidae is expanding while that of Gryllidae is shrinking (Yuan et al. 2021). As shown in Fig. 1, these two clades are the species-richest clade in Ensifera, indicating extensive species divergence. Yuan et al. have proposed the following scenario for the divergence of Tettigoniidae and Gryllidae in relation to their genome size (Yuan et al. 2021). The increase in the number of species in the Tettigoniidae may be due to events such as gene duplications and transposon insertions, which are also responsible for genome size expansion, causing beneficial mutations that result in the acquisition of new ecological niches (Dufresne and Jeffery 2011). In contrast, the reduction of genome size in Gryllidae may be advantageous for survival by saving extra metabolic costs (Gregory and Hebert 1999). One interesting study using the bow-winged grasshopper *Chorthippus biguttulus* investigated the relationship between male genome size and female preference for songs (Schielzeth et al. 2014). This study found that genome size is reflected in male courtship songs and that females prefer the songs of males with smaller genome sizes (Schielzeth et al. 2014). Investigating the ecological effects of genome size reduction in Gryllidae will be a topic of future research.

## Cricket as an alternative animal protein resource in the future

Orthopteran insects, including locusts, grasshoppers, and crickets, are consumed and occasionally reared, worldwide. Among orthopterans, cricket is considered one of the insects that are attracting attention most intensively as a source of food for humans and feed for livestock and to have the potential to contribute to food security and alleviate malnutrition. Indeed, among orthopterans, crickets are by far the most consumed insect in the world (Oonincx et al. 2015). As mentioned in the Introduction section, crickets have many advantages such as their high growth efficiency and trait that allows for high-density breeding (Nagasaki and Defoliart 1991; Oonincx et al. 2015). It is also worth noting that crickets are omnivorous, which provides the potential to feed on various types of food and agricultural wastes. Moreover, genetic modification techniques have already been established for crickets. These technologies include *piggyBac*-mediated transformation, TALEN, and CRISPR-Cas9 system (Shinmyo et al. 2004; Watanabe et al. 2012; Awata et al. 2015). These characteristics have made crickets a promising alternative animal protein source for researchers around the world.

Various crickets have been consumed in Asia, Latin America, and Africa since prehistoric times. Nowadays, a wide variety of Gryllidea insects are being consumed and reared around the world. 62 species of crickets have been reported to be consumed as human food or be used as livestock feed around the world (Magara et al. 2021). Representative edible species include *Gryllus bimaculatus*, *G. similis*, *G. assimilis*, *Gryllodes sigillatus*, *Acheta domesticus*,* Brachytrupes* sp., and *Gryllotalpa* sp. The most frequently consumed family of crickets worldwide is the family Gryllidae, followed by Gryllotalpidae (Jongema 2017). It has been recorded that as many as 20,000 farmers in Thailand produced 7500 tons of crickets each year (Hanboonsong et al. 2013). In Bangladesh, *Brachytrupes portentosus*, the largest species of cricket, is collected from the wild and sold in local markets (Hasan et al. 2021). In some places, this species is also consumed as a supplement for healthy mental development and pre- and postnatal care (Rajkhowa and Rokozeno 2016).

There are many cases of farming crickets in different corners of the world, but keeping insects in a confinement often has a genetic impact through inbreeding depression, for instance, and hence new crickets in the wild are added to keep the farming. The inbreeding depression apparently is one of the causes that prevents crickets from being domesticated and from expanding their production that sustains entomophagy. The cause of inbreeding depression lies predominantly in the presence of recessive mutations. Genome sequences can provide the basis for precise genomic breeding. Once these recessive mutations in genes are identified based on the genome sequences, new ways to circumvent the inbreeding depression can be figured out. Cricket farming and breeding based on their genomic information will in effect mitigate food insecurity and contribute to the livelihood of mankind.

Recent technology applied for plants can be used for crickets to expand their usage as food and feed. In plants, recent progress in genome science and genome editing technologies has sparked a revolution in the field of precision breeding. The accessibility of high-throughput sequencing technologies and computational analysis has led to the rapid accumulation of genomic data. The genomic information can be used to identify desirable genes and traits that can be incorporated into wild relatives of crop plants. Combining these data with genome editing technologies such as CRISPR-Cas9, it is now possible to edit plant genomes with extreme precision. Recent technology of genome editing has relatively easily enabled the manipulation of traits with a polygenic basis (Zsögön et al. 2018). We expect that the application of the latest biophysical technologies to crickets can realize the domestication of the species and overcome the challenges of commercialization of crickets as food and feed, such as size, taste, and allergies.

## Conclusion and perspective

Advances in biophysical technologies in the last few decades have generated a large number of genomic datasets derived from a wide variety of insects, which has brought entomology into a new era and is making progress even in the field of applied research including entomophagy. Crickets have been used as an experimental model of hemimetabolous insects for developmental biology and neuroscience, which is due to the fact that crickets have the following characteristics: (1) relatively short life cycle with about 1 month to hatch; (2) easy to maintain populations in the laboratory; and (3) capable of genetic manipulation by RNAi or CRISPR-Cas9. The genomic information of crickets will not only provide insight into the genetic background underlying their ecological diversity but will also shed light on the evolution of genome size in insects and TE-driven evolution. In addition, the information will help the domestication of crickets for global food security.

## Supplementary Information

Below is the link to the electronic supplementary material.

Supplementary file1 (XLSX 1726 KB)

## References

[CR1] Adams MD, Celniker SE, Holt RA, Evans CA, Gocayne JD, Amanatides PG, Scherer SE, Li PW, Hoskins RA, Galle RF, George RA, Lewis SE, Richards S, Ashburner M, Henderson SN, Sutton GG, Wortman JR, Yandell MD, Zhang Q, Chen LX, Brandon RC, Rogers YH, Blazej RG, Champe M, Pfeiffer BD, Wan KH, Doyle C, Baxter EG, Helt G, Nelson CR, Gabor GL, Abril JF, Agbayani A, An HJ, Andrews-Pfannkoch C, Baldwin D, Ballew RM, Basu A, Baxendale J, Bayraktaroglu L, Beasley EM, Beeson KY, Benos PV, Berman BP, Bhandari D, Bolshakov S, Borkova D, Botchan MR, Bouck J, Brokstein P, Brottier P, Burtis KC, Busam DA, Butler H, Cadieu E, Center A, Chandra I, Cherry JM, Cawley S, Dahlke C, Davenport LB, Davies P, de Pablos B, Delcher A, Deng Z, Mays AD, Dew I, Dietz SM, Dodson K, Doup LE, Downes M, Dugan-Rocha S, Dunkov BC, Dunn P, Durbin KJ, Evangelista CC, Ferraz C, Ferriera S, Fleischmann W, Fosler C, Gabrielian AE, Garg NS, Gelbart WM, Glasser K, Glodek A, Gong F, Gorrell JH, Gu Z, Guan P, Harris M, Harris NL, Harvey D, Heiman TJ, Hernandez JR, Houck J, Hostin D, Houston KA, Howland TJ, Wei MH, Ibegwam C, Jalali M, Kalush F, Karpen GH, Ke Z, Kennison JA, Ketchum KA, Kimmel BE, Kodira CD, Kraft C, Kravitz S, Kulp D, Lai Z, Lasko P, Lei Y, Levitsky AA, Li J, Li Z, Liang Y, Lin X, Liu X, Mattei B, McIntosh TC, McLeod MP, McPherson D, Merkulov G, Milshina NV, Mobarry C, Morris J, Moshrefi A, Mount SM, Moy M, Murphy B, Murphy L, Muzny DM, Nelson DL, Nelson DR, Nelson KA, Nixon K, Nusskern DR, Pacleb JM, Palazzolo M, Pittman GS, Pan S, Pollard J, Puri V, Reese MG, Reinert K, Remington K, Saunders RD, Scheeler F, Shen H, Shue BC, Sidén-Kiamos I, Simpson M, Skupski MP, Smith T, Spier E, Spradling AC, Stapleton M, Strong R, Sun E, Svirskas R, Tector C, Turner R, Venter E, Wang AH, Wang X, Wang ZY, Wassarman DA, Weinstock GM, Weissenbach J, Williams SM, WoodageT WKC, Wu D, Yang S, Yao QA, Ye J, Yeh RF, Zaveri JS, Zhan M, Zhang G, Zhao Q, Zheng L, Zheng XH, Zhong FN, Zhong W, Zhou X, Zhu S, Zhu X, Smith HO, Gibbs RA, Myers EW, Rubin GM, Venter JC (2000). The genome sequence of *Drosophila melanogaster*. Science.

[CR2] Alfsnes K, Leinaas HP, Hessen DO (2017). Genome size in arthropods; different roles of phylogeny, habitat and life history in insects and crustaceans. Ecol Evol.

[CR3] Allegrucci G, Trewick SA, Fortunato A, Carchini G, Sbordoni V (2010). Cave crickets and cave weta (Orthoptera, Rhaphidophoridae) from the southern end of the world: a molecular phylogeny test of biogeographical hypotheses. J Orthoptera Res.

[CR4] Andrés JA, Larson EL, Bogdanowicz SM, Harrison RG (2013). Patterns of transcriptome divergence in the male accessory gland of two closely related species of field crickets. Genetics.

[CR5] Awata H, Watanabe T, Hamanaka Y, Mito T, Noji S, Mizunami M (2015). Knockout crickets for the study of learning and memory: dopamine receptor Dop1 mediates aversive but not appetitive reinforcement in crickets. Sci Rep.

[CR6] Bando T, Ishimaru Y, Kida T, Hamada Y, Matsuoka Y, Nakamura T, Ohuchi H, Noji S, Mito T (2013). Analysis of RNA-Seq data reveals involvement of JAK/STAT signalling during leg regeneration in the cricket *Gryllus bimaculatus*. Development.

[CR7] Bao W, Kojima KK, Kohany O (2015). Repbase Update, a database of repetitive elements in eukaryotic genomes. Mob DNA.

[CR8] Beçak ML, Denaro L, Beçak W (1970). Polyploidy and mechanisms of karyotypic diversification in Amphibia. Cytogenet Genome Res.

[CR9] Beccaloni GW (2014) Cockroach Species File Online. Version 5.0/5.0. http://Cockroach.SpeciesFile.org. Accessed 25 Aug 2021

[CR10] Bennett MD (1977). The time and duration of meiosis. Philos Trans R Soc Lond B Biol Sci.

[CR11] Berdan EL, Blankers T, Waurick I, Mazzoni CJ, Mayer F (2016). A genes eye view of ontogeny: de novo assembly and profiling of the *Gryllus **rubens* transcriptome. Mol Ecol Resour.

[CR12] Blackmon H, Demuth JP (2015). The fragile Y hypothesis: Y chromosome aneuploidy as a selective pressure in sex chromosome and meiotic mechanism evolution. BioEssays.

[CR13] Blackmon H, Ross L, Bachtrog D (2017). Sex determination, sex chromosomes, and karyotype evolution in insects. J Hered.

[CR14] Blankers T, Oh KP, Bombarely A, Shaw KL (2018). The genomic architecture of a rapid island radiation: recombination rate variation, chromosome structure, and genome assembly of the Hawaiian cricket Laupala. Genetics.

[CR15] Blankers T, Vilaça ST, Waurick I, Gray DA, Hennig RM, Mazzoni CJ, Mayer F, Berdan EL (2018). Demography and selection shape transcriptomic divergence in field crickets. Evolution.

[CR16] Blankers T, Oh KP, Shaw KL (2019a) Parallel genomic architecture underlies repeated sexual signal divergence in Hawaiian Laupala crickets. Proc Biol Sci 286:20191479. 10.1098/rspb.2019.1479 10.1098/rspb.2019.1479 PMC6790767 31594503

[CR17] Blankers T, Berdan EL, Hennig RM, Mayer F (2019). Physical linkage and mate preference generate linkage disequilibrium for behavioral isolation in two parapatric crickets. Evolution.

[CR18] Bonasio R, Li Q, Lian J, Mutti NS, Jin L, Zhao H, Zhang P, Wen P, Xiang H, Ding Y, Jin Z, Shen SS, Wang Z, Wang W, Wang J, Berger SL, Liebig J, Zhang G, Reinberg D (2012). Genome-wide and caste-specific DNA methylomes of the ants *Camponotus floridanus* and *Harpegnathos saltator*. Curr Biol.

[CR19] Bourque G, Burns KH, Gehring M, Gorbunova V, Seluanov A, Hammell M, Imbeault M, Izsvák Z, Levin HL, Macfarlan TS, Mager DL, Feschotte C (2018). Ten things you should know about transposable elements. Genome Biol.

[CR20] Brock PD, Büscher T, Baker E (2018) Phasmida Species File Online. Version 5.0/5.0. http://Phasmida.SpeciesFile.org. Accessed 25 Aug 2021

[CR21] Bucheton A, Paro R, Sang HM, Pelisson A, Finnegan DJ (1984). The molecular basis of I-R hybrid dysgenesis in Drosophila melanogaster: identification, cloning, and properties of the I factor. Cell.

[CR22] Carbone L, Harris RA, Gnerre S, Veeramah KR, Lorente-Galdos B, Huddleston J, Meyer TJ, Herrero J, Roos C, Aken B, Anaclerio F, Archidiacono N, Baker C, Barrell D, Batzer MA, Beal K, Blancher A, Bohrson CL, Brameier M, Campbell MS, Capozzi O, Casola C, Chiatante G, Cree A, Damert A, de Jong PJ, Dumas L, Fernandez-Callejo M, Flicek P, Fuchs NV, Gut I, Gut M, Hahn MW, Hernandez-Rodriguez J, Hillier LW, Hubley R, Ianc B, Izsvák Z, Jablonski NG, Johnstone LM, Karimpour-Fard A, Konkel MK, Kostka D, Lazar NH, Lee SL, Lewis LR, Liu Y, Locke DP, Mallick S, Mendez FL, Muffato M, Nazareth LV, Nevonen KA, O’Bleness M, Ochis C, Odom DT, Pollard KS, Quilez J, Reich D, Rocchi M, Schumann GG, Searle S, Sikela JM, Skollar G, Smit A, Sonmez K, ten Hallers B, Terhune E, Thomas GW, Ullmer B, Ventura M, Walker JA, Wall JD, Walter L, Ward MC, Wheelan SJ, Whelan CW, White S, Wilhelm LJ, Woerner AE, Yandell M, Zhu B, Hammer MF, Marques-Bonet T, Eichler EE, Fulton L, Fronick C, Muzny DM, Warren WC, Worley KC, Rogers J, Wilson RK, Gibbs RA (2014). Gibbon genome and the fast karyotype evolution of small apes. Nature.

[CR23] Cavalier-Smith T, Cavalier-Smith T (1985). Cell volume and the evolution of eukaryote genome size. The Evolution of Genome Size.

[CR24] Chen S, Li X (2007). Transposable elements are enriched within or in close proximity to xenobiotic-metabolizing cytochrome P450 genes. BMC Evol Biol.

[CR25] Chintauan-Marquier IC, Legendre F, Hugel S, Robillard T, Grandcolas P, Nel A, Zuccon D, Desutter-Grandcolas L (2016). Laying the foundations of evolutionary and systematic studies in crickets (Insecta, Orthoptera): a multilocus phylogenetic analysis. Cladistics.

[CR26] Cičková H, Newton GL, Lacy RC, Kozánek M (2015). The use of fly larvae for organic waste treatment. Waste Manag.

[CR27] Coello Alvarado LE, MacMillan HA, Sinclair BJ (2015). Chill-tolerant *Gryllus* crickets maintain ion balance at low temperatures. J Insect Physiol.

[CR28] d’Alencon E, Sezutsu H, Legeai F, Permal E, Bernard-Samain S, Gimenez S, Gagneur C, Cousserans F, Shimomura M, Brun-Barale A (2010). Extensive synteny conservation of holocentric chromosomes in Lepidoptera despite high rates of local genome rearrangements. Proc Natl Acad Sci USA.

[CR29] Davidowitz G, D’Amico LJ, Nijhout HF (2004). The effects of environmental variation on a mechanism that controls insect body size. Evol Ecol Res.

[CR30] Des Marteaux LE, McKinnon AH, Udaka H, Toxopeus J, Sinclair BJ (2017). Effects of cold-acclimation on gene expression in fall field cricket (*Gryllus pennsylvanicus*) ionoregulatory tissues. BMC Genomics.

[CR31] de Vos JM, Augustijnen H, Bätscher L, Lucek K (2020) Speciation through chromosomal fusion and fission in Lepidoptera. Philos Trans R Soc Lond B Biol Sci 375:20190539. 10.1098/rstb.2019.0539 10.1098/rstb.2019.0539 PMC7423279 32654638

[CR32] DeWalt RE, Maehr MD, Hopkins H, Neu-Becker U, Stueber G (2021) Plecoptera Species File Online. Version 5.0/5.0. http://Plecoptera.SpeciesFile.org. Accessed 25 Aug 2021

[CR33] Dirsh VM (1954). Lathicerinae, a new subfamily of Acrididae (Orthoptera). Ann Mag Nat Hist.

[CR34] Dolezel J, Bartos J, Voglmayr H, Greilhuber J (2003). Nuclear DNA content and genome size of trout and human. Cytometry A.

[CR35] Donoughe S, Extavour CG (2016). Embryonic development of the cricket Gryllus bimaculatus. Dev Biol.

[CR36] Doyle JJ, Flagel LE, Paterson AH, Rapp RA, Soltis DE, Soltis PS, Wendel JF (2008). Evolutionary genetics of genome merger and doubling in plants. Annu Rev Genet.

[CR37] Dufresne F, Jeffery N (2011). A guided tour of large genome size in animals: what we know and where we are heading. Chromosome Res.

[CR38] Dumont BL (2017). Variation and evolution of the meiotic requirement for crossing over in mammals. Genetics.

[CR39] Eades DC, Otte D, Cigliano MM, Braun H (2014) Orthoptera Species File. Version 5.0/5.0. http://Orthoptera.SpeciesFile.org. Accessed 18 Aug 2021

[CR40] Emmel TC, Eliazar PJ, Brown KS, Suomalainen E, Scriber JM, Tsubaki Y, Lederhouse RC (1995). Chromosome evolution in the Papilionidae. Swallowtail butterflies: their ecology and evolution.

[CR41] Feng S, Cokus SJ, Zhang X, Chen PY, Bostick M, Goll MG, Hetzel J, Jain J, Strauss SH, Halpern ME, Ukomadu C, Sadler KC, Pradhan S, Pellegrini M, Jacobsen SE (2010). Conservation and divergence of methylation patterning in plants and animals. Proc Natl Acad Sci USA.

[CR42] Finnegan DJ (1989). Eukaryotic transposable elements and genome evolution. Trend Genet.

[CR43] Fisher HP, Pascual MG, Jimenez SI, Michaelson DA, Joncas CT, Quenzer ED, Christie AE, Horch HW (2018) *De novo* assembly of a transcriptome for the cricket *Gryllus bimaculatus* prothoracic ganglion: an invertebrate model for investigating adult central nervous system compensatory plasticity. PLoS One 13:e0199070. 10.1371/journal.pone.0199070 10.1371/journal.pone.0199070 PMC6040699 29995882

[CR44] Frank JH, Parkman JP (1999). Integrated pest management of pest mole crickets with emphasis on the Southeastern USA. Integrated Pest Manag Rev.

[CR45] Gilbert C, Peccoud J, Cordaux R (2021). Transposable elements and the evolution of insects. Annu Rev Entomol.

[CR46] Godfray HC, Beddington JR, Crute IR, Haddad L, Lawrence D, Muir JF, Pretty J, Robinson S, Thomas SM, Toulmin C (2010). Food security: the challenge of feeding 9 billion people. Science.

[CR47] González J, Karasov TL, Messer PW, Petrov DA (2010) Genome-wide patterns of adaptation to temperate environments associated with transposable elements in Drosophila. PLoS Genet 6:1000905. 10.1371/journal.pgen.1000905 10.1371/journal.pgen.1000905 PMC2851572 20386746

[CR48] Gordon JL, Byrne KP, Wolfe KH (2011) Mechanisms of chromosome number evolution in yeast. PLoS Genet 7:e1002190. 10.1371/journal.pgen.1002190 10.1371/journal.pgen.1002190 PMC3141009 21811419

[CR49] Gray DA, Huang H, Knowles LL (2008). Molecular evidence of a peripatric origin for two sympatric species of field crickets (*Gryllus rubens* and *G. texensis*) revealed from coalescent simulations and population genetic tests. Mol Ecol.

[CR50] Gregory TR, Hebert PD (1999). The modulation of DNA content: proximate causes and ultimate consequences. Genome Res.

[CR51] Gregory TR, Hebert PD, Kolasa J (2000). Evolutionary implications of the relationship between genome size and body size in flatworms and copepods. Heredity.

[CR52] Gregory TR (2002). A bird’s-eye view of the C-value enigma: genome size, cell size, and metabolic rate in the class aves. Evolution.

[CR53] Gregory TR (2021) Animal Genome Size Database. http://www.genomesize.com. Accessed 25 Aug 2021

[CR54] Greilhuber J, Dolezel J, Lysák MA, Bennett MD (2005). The origin, evolution and proposed stabilization of the terms “genome size” and “C-value” to describe nuclear DNA contents. Ann Bot.

[CR55] Grimaldi D, Engel MS (2005). Evolution of the insects.

[CR56] Gupta YM, Tanasarnpaiboon S, Buddhachat K, Peyachoknagul S, Inthim P, Homchan S (2020) Development of microsatellite markers for the house cricket, *Acheta domesticus* (Orthoptera: Gryllidae). Biodiversitas 21:4094–4099. 10.13057/biodiv/d210921

[CR57] Hanboonsong Y, Jamjanya T, Durst PB (2013). Six-legged livestock: edible insect farming, collection and marketing in Thailand.

[CR58] Hanrahan SJ, Johnston JS (2011). New genome size estimates of 134 species of arthropods. Chromosome Res.

[CR59] Hasan MM, Rahman MM, Kataoka K, Yura K, Faruque MO, Shadhen FR, Mondal MF (2021) Edible wild field cricket (Brachytrupes portentosus) trading in Bangladesh. J Insects Food Feed 7:1255-1262. 10.3920/JIFF2020.0163

[CR60] Hill A, Zheng X, Li X, McKinney R, Dickman D, Ben-Shahar Y (2017). The *Drosophila* postsynaptic DEG/ENaC channel *ppk29* contributes to excitatory neurotransmission. J Neurosci.

[CR61] Hill J, Rastas P, Hornett EA, Neethiraj R, Clark N, Morehouse N, de la Paz Celorio-Mancera M, Cols JC, Dircksen H, Meslin C, Keehnen N, Pruisscher P, Sikkink K, Vives M, Vogel H, Wiklund C, Woronik A, Boggs CL, Nylin S, Wheat CW (2019) Unprecedented reorganization of holocentric chromosomes provides insights into the enigma of lepidopteran chromosome evolution. Sci Adv 5:eaau3648. 10.1126/sciadv.aau3648 10.1126/sciadv.aau3648 PMC6561736 31206013

[CR62] Hopkins H, Maehr MD (2018) Grylloblattodea Species File. Version 5.0/5.0. http://Grylloblattodea.SpeciesFile.org. Accessed 25 Aug 2021

[CR63] Hopkins H, Maehr MD, Haas F, Deem LS (2018) Dermaptera Species File. Version 5.0/5.0. http://Dermaptera.SpeciesFile.org. Accessed 25 Aug 2021

[CR64] Horch HW, Sheldon E, Cutting CC, Williams CR, Riker DM, Peckler HR, Sangal RB (2011). Bilateral consequences of chronic unilateral deafferentation in the auditory system of the cricket *Gryllus bimaculatus*. Dev Neurosci.

[CR65] Hotaling S, Sproul JS, Heckenhauer J, Powell A, Larracuente AM, Pauls SU, Kelley JL, Frandsen PB (2021) Long reads are revolutionizing 20 years of insect genome sequencing. Genome Biol Evol 13:evab138. 10.1093/gbe/evab138 10.1093/gbe/evab138 PMC8358217 34152413

[CR66] Hussain A, Ali MW, AlJabr AM, Al-Kahtani SN (2020). Insights into the *Gryllus bimaculatus* immune-related transcriptomic profiling to combat naturally invading pathogens. J Fungi (basel).

[CR67] Ishimaru Y, Tomonari S, Matsuoka Y, Watanabe T, Miyawaki K, Bando T, Tomioka K, Ohuchi H, Noji S, Mito T (2016). TGF-β signaling in insects regulates metamorphosis via juvenile hormone biosynthesis. Proc Natl Acad Sci USA.

[CR68] Itokawa K, Komagata O, Kasai S, Okamura Y, Masada M, Tomita T (2010). Genomic structures of Cyp9m10 in pyrethroid resistant and susceptible strains of *Culex quinquefasciatus*. Insect Biochem Mol Biol.

[CR69] Jongema Y (2017). List of Edible Insects of the World.

[CR70] Kataoka K, Minei R, Ide K, Ogura A, Takeyama H, Takeda M, Suzuki T, Yura K, Asahi T (2020). The draft genome dataset of the Asian cricket *Teleogryllus occipitalis* for molecular research toward entomophagy. Front Genet.

[CR71] Kidwell MG (2002). Transposable elements and the evolution of genome size in eukaryotes. Genetica.

[CR72] Kim YB, Oh JH, McIver LJ, Rashkovetsky E, Michalak K, Garner HR, Kang L, Nevo E, Korol AB, Michalak P (2014). Divergence of *Drosophila melanogaster* repeatomes in response to a sharp microclimate contrast in Evolution Canyon Israel. Proc Natl Acad Sci USA.

[CR73] Knowles LL (2001). Genealogical portraits of speciation in montane grasshoppers (genus Melanoplus) from the sky islands of the Rocky Mountains. Proc Royal Soc B.

[CR74] Komatsu T, Maruyama M, Ueda S, Itino T (2008). mtDNA phylogeny of Japanese ant crickets (Orthoptera: Myrmecophilidae): diversification in host specificity and habitat use. Sociobiology.

[CR75] Kono N, Nakamura H, Ohtoshi R, Arakawa K (2021) Transcriptomic data during development of a two-spotted cricket *Gryllus bimaculatus*. Data Brief 38:107388. 10.1016/j.dib.2021.107388 10.1016/j.dib.2021.107388 PMC8473666 34604480

[CR76] Larson EL, Andrés JA, Bogdanowicz SM, Harrison RG (2013). Differential introgression in a mosaic hybrid zone reveals candidate barrier genes. Evolution.

[CR77] Larson EL, White TA, Ross CL, Harrison RG (2014). Gene flow and the maintenance of species boundaries. Mol Ecol.

[CR78] Li Z, Tiley GP, Galuska SR, Reardon CR, Kidder TI, Rundell RJ, Barker MS (2018). Multiple large-scale gene and genome duplications during the evolution of hexapods. Proc Natl Acad Sci USA.

[CR79] Lima-de-Faria A, Gustafsson T, Jaworska H (1973). Amplification of ribosomal DNA in Acheta. II. The number of nucleotide pairs of the chromosomes and chromomeres involved in amplification. Hereditas.

[CR80] Lukhtanov VA, Dincă V, Talavera G, Vila R (2011). Unprecedented within-species chromosome number cline in the Wood White butterfly Leptidea sinapis and its significance for karyotype evolution and speciation. BMC Evol Biol.

[CR81] Lukhtanov VA (2015). The blue butterfly *Polyommatus* (*Plebicula*) *atlanticus* (Lepidoptera, Lycaenidae) holds the record of the highest number of chromosomes in the non-polyploid eukaryotic organisms. Comp Cytogenet.

[CR82] Lukhtanov VA, Dincă V, Friberg M, Šíchová J, Olofsson M, Vila R, Marec F, Wiklund C (2018). Versatility of multivalent orientation, inverted meiosis, and rescued fitness in holocentric chromosomal hybrids. Proc Natl Acad Sci USA.

[CR83] Maehr MD, Hopkins H (2017) Mantophasmatodea Species File. Version 5.0/5.0. http://Mantophasmatodea.SpeciesFile.org. Accessed 25 Aug 2021

[CR84] Maehr MD, Hopkins H (2018) Zoraptera Species File. Version 5.0/5.0. http://Zoraptera.SpeciesFile.org. Accessed 25 Aug 2021

[CR85] Maehr MD, Hopkins H, Eades DC (2018) Embioptera Species File Online. Version 5.0/5.0. http://Embioptera.SpeciesFile.org. Accessed 25 Aug 2021

[CR86] Magara HJO, Niassy S, Ayieko MA, Mukundamago M, Egonyu JP, Tanga CM, Kimathi EK, Ongere JO, Fiaboe KKM, Hugel S, Orinda MA, Roos N, Ekesi S (2021) Edible crickets (Orthoptera) around the world: distribution, nutritional value, and other benefits—a review. Front Nutr 7:537915. 10.3389/fnut.2020.537915 10.3389/fnut.2020.537915 PMC7835793 33511150

[CR87] Makino S (1931). The chromosomes of *Diestrammena japonica* KARNY (an orthopteran). Dobutsugaku-Zasshi.

[CR88] Maroja LS, Andrés JA, Harrison RG (2009). Genealogical discordance and patterns of introgression and selection across a cricket hybrid zone. Evolution.

[CR89] Maroja LS, Larson EL, Bogdanowicz SM, Harrison RG (2015) Genes with restricted introgression in a field cricket (*Gryllus firmus*/*Gryllus pennsylvanicus*) hybrid zone are concentrated on the X chromosome and a single autosome. G3 (Bethesda) 5:2219–2227. 10.1534/g3.115.021246 10.1534/g3.115.021246 PMC4632042 26311650

[CR90] Masaki S, Ohmachi F (1967). Divergence of photoperidodic response and hybrid develoment in Teleogryllus (Orthoptera : Gryllidae). Kontyu.

[CR91] Matsumoto Y, Matsumoto CS, Mizunami M (2018). Signaling pathways for long-term memory formation in the cricket. Front Psychol.

[CR92] Meier T, Reichert H (1990). Embryonic development and evolutionary origin of the orthopteran auditory organs. J Neurobiol.

[CR93] Misof B, Liu S, Meusemann K, Peters RS, Donath A, Mayer C, Frandsen PB, Ware J, Flouri T, Beutel RG, Niehuis O, Petersen M, Izquierdo-Carrasco F, Wappler T, Rust J, Aberer AJ, Aspöck U, Aspöck H, Bartel D, Blanke A, Berger S, Böhm A, Buckley TR, Calcott B, Chen J, Friedrich F, Fukui M, Fujita M, Greve C, Grobe P, Gu S, Huang Y, Jermiin LS, Kawahara AY, Krogmann L, Kubiak M, Lanfear R, Letsch H, Li Y, Li Z, Li J, Lu H, Machida R, Mashimo Y, Kapli P, McKenna DD, Meng G, Nakagaki Y, Navarrete-Heredia JL, Ott M, Ou Y, Pass G, Podsiadlowski L, Pohl H, von Reumont BM, Schütte K, Sekiya K, Shimizu S, Slipinski A, Stamatakis A, Song W, Su X, Szucsich NU, Tan M, Tan X, Tang M, Tang J, Timelthaler G, Tomizuka S, Trautwein M, Tong X, Uchifune T, Walzl MG, Wiegmann BM, Wilbrandt J, Wipfler B, Wong TK, Wu Q, Wu G, Xie Y, Yang S, Yang Q, Yeates DK, Yoshizawa K, Zhang Q, Zhang R, Zhang W, Zhang Y, Zhao J, Zhou C, Zhou L, Ziesmann T, Zou S, Li Y, Xu X, Zhang Y, Yang H, Wang J, Wang J, Kjer KM, Zhou X (2014). Phylogenomics resolves the timing and pattern of insect evolution. Science.

[CR94] Mito T, Noji S (2008) The two-spotted cricket Gryllus bimaculatus: an emerging model for developmental and regeneration studies. CSH Protoc 2008:pdb.emo110. 10.1101/pdb.emo110 10.1101/pdb.emo110 21356736

[CR95] Moran PA, Pascoal S, Cezard T, Risse JE, Ritchie MG, Bailey NW (2018). Opposing patterns of intraspecific and interspecific differentiation in sex chromosomes and autosomes. Mol Ecol.

[CR96] Moretti A, Sabato S (1984). Karyotype evolution by centromeric fission in Zamia (Cycadales). Plant Syst Evol.

[CR97] Nagasaki BJ, Defoliart GR (1991). Comparison of diets for mass-rearing *Acheta domesticus* (Orthoptera: Gryllidae) as a novelty food, and comparison of food conversion efficiency with values reported for livestock. J Econ Entomol.

[CR98] Nakamura T, Mito T, Bando T, Ohuchi H, Noji S (2008). Dissecting insect leg regeneration through RNA interference. Cell Mol Life Sci.

[CR99] Nanoth Vellichirammal N, Zera AJ, Schilder RJ, Wehrkamp C, Riethoven JJ, Brisson JA (2014) *De novo* transcriptome assembly from fat body and flight muscles transcripts to identify morph-specific gene expression profiles in *Gryllus firmus*. PLoS One 9:e82129. 10.1371/journal.pone.0082129 10.1371/journal.pone.0082129 PMC3885399 24416137

[CR100] Nellemann C, MacDevette M, Manders T, Eickhout B, Svihus B, Prins AG (2009). The environmental food crisis-the environment’s role in averting future food crises. A UNEP Rapid Response Assessment.

[CR101] Oonincx DGAB, van Broekhoven S, van Huis A, van Loon JJA (2015) Feed conversion, survival and development, and composition of four insect species on diets composed of food by-products. PLoS ONE 10:e0144601. 10.1371/journal.pone.0144601 10.1371/journal.pone.0144601 PMC4689427 26699129

[CR102] Oppert B, Perkin LC, Lorenzen M, Dossey AT (2020). Transcriptome analysis of life stages of the house cricket, *Acheta domesticus*, to improve insect crop production. Sci Rep.

[CR103] Otte D, Spearman L, Martin BD (2017) Mantodea species file online. Version 5.0/5.0. http://Mantodea.SpeciesFile.org. Accessed 25 Aug 2021

[CR104] Prasad MP, Detchou DKE, Wang F, Ledwidge LL, Kingston SE, Wilson Horch H (2021). Transcriptional expression changes during compensatory plasticity in the terminal ganglion of the adult cricket *Gryllus bimaculatus*. BMC Genomics.

[CR105] Pascoal S, Cezard T, Eik-Nes A, Gharbi K, Majewska J, Payne E, Ritchie MG, Zuk M, Bailey NW (2014). Rapid convergent evolution in wild crickets. Curr Biol.

[CR106] Pascoal S, Liu X, Fang Y, Paterson S, Ritchie MG, Rockliffe N, Zuk M, Bailey NW (2018). Increased socially mediated plasticity in gene expression accompanies rapid adaptive evolution. Ecol Lett.

[CR107] Pascoal S, Risse JE, Zhang X, Blaxter M, Cezard T, Challis RJ, Gharbi K, Hunt J, Kumar S, Langan E, Liu X, Rayner JG, Ritchie MG, Snoek BL, Trivedi U, Bailey NW (2019). Field cricket genome reveals the footprint of recent, abrupt adaptation in the wild. Evol Lett.

[CR108] Petersen M, Armisén D, Gibbs RA, Hering L, Khila A, Mayer G, Richards S, Niehuis O, Misof B (2019). Diversity and evolution of the transposable element repertoire in arthropods with particular reference to insects. BMC Evol Biol.

[CR109] Rainford JL, Hofreiter M, Mayhew PJ (2016). Phylogenetic analyses suggest that diversification and body size evolution are independent in insects. BMC Evol Biol.

[CR110] Rajkhowa D, Rokozeno DMK (2016). Insect-based medicine: a review of present status and prospects of entomo-therapeutic. IJAEB.

[CR111] Rayner JG, Hitchcock TJ, Bailey NW (2021) Variable dosage compensation is associated with female consequences of an X-linked, male-beneficial mutation. Proc Biol Sci 288:20210355. 10.1098/rspb.2021.0355 10.1098/rspb.2021.0355 PMC8059673 33757350

[CR112] Roff DA (2000). Trade-offs between growth and reproduction: an analysis of the quantitative genetic evidence. J Evol Biol.

[CR113] Román-Palacios C, Medina CA, Zhan SH, Barker MS (2021). Animal chromosome counts reveal a similar range of chromosome numbers but with less polyploidy in animals compared to flowering plants. J Evol Biol.

[CR114] Rubin E, Lithwick G, Levy AA (2001) Structure and evolution of the *hAT* transposon superfamily. Genetics 158:949–957. 10.1093/genetics/158.3.949 10.1093/genetics/158.3.949 PMC1461711 11454746

[CR115] Ruckman SN, Jonika MM, Casola C, Blackmon H (2020) Chromosome number evolves at equal rates in holocentric and monocentric clades. PLoS Genet 16:e1009076. 10.1371/journal.pgen.1009076 10.1371/journal.pgen.1009076 PMC7584213 33048946

[CR116] Sanno R, Kataoka K, Hayakawa S, Ide K, Nguyen CN, Nguyen TP, Le BTN, Kim OTP, Mineta K, Takeyama H, Takeda M, Sato T, Suzuki T, Yura K, Asahi T (2021) Comparative analysis of mitochondrial genomes in Gryllidea (Insecta: Orthoptera): implications for adaptive evolution in ant-loving crickets. Genome Biol Evol 13:evab222. 10.1093/gbe/evab222 10.1093/gbe/evab222 PMC8511664 34554226

[CR117] Satoh A, Yoshioka E, Numata H (2008). Circatidal activity rhythm in the mangrove cricket *Apteronemobius asahinai*. Biol Lett.

[CR118] Satoh A, Terai Y (2019). Circatidal gene expression in the mangrove cricket *Apteronemobius asahinai*. Sci Rep.

[CR119] Satoh A, Takasu M, Yano K, Terai Y (2021). *De novo* assembly and annotation of the mangrove cricket genome. BMC Res Notes.

[CR120] Schielzeth H, Streitner C, Lampe U, Franzke A, Reinhold K (2014). Genome size variation affects song attractiveness in grasshoppers: evidence for sexual selection against large genomes. Evolution.

[CR121] Sessegolo C, Burlet N, Haudry A (2016) Strong phylogenetic inertia on genome size and transposable element content among 26 species of flies. Biol Lett 12:20160407. 10.1098/rsbl.2016.0407 10.1098/rsbl.2016.0407 PMC5014035 27576524

[CR122] Shinmyo Y, Mito T, Matsushita T, Sarashina I, Miyawaki K, Ohuchi H, Noji S (2004). piggyBac-mediated somatic transformation of the two-spotted cricket, *Gryllus bimaculatus*. Dev Growth Differ.

[CR123] Smit A, Hubley R, Grenn P (2015) RepeatMasker Open-4.0. http://www.repeatmasker.org

[CR124] Sun L, Johnson AF, Donohue RC, Li J, Cheng J, Birchler JA (2013). Dosage compensation and inverse effects in triple X metafemales of Drosophila. Proc Natl Acad Sci USA.

[CR125] Song H, Amédégnato C, Cigliano MM, Desutter-Grandcolas L, Heads SW, Huang Y, Otte D, Whiting MF (2015). 300 million years of diversification: elucidating the patterns of orthopteran evolution based on comprehensive taxon and gene sampling. Cladistics.

[CR126] Song H (2018). Biodiversity of orthoptera. Insect Biodivers Sci Soc.

[CR127] Song H, Béthoux O, Shin S, Donath A, Letsch H, Liu S, McKenna DD, Meng G, Misof B, Podsiadlowski L, Zhou X, Wipfler B, Simon S (2020). Phylogenomic analysis sheds light on the evolutionary pathways towards acoustic communication in Orthoptera. Nat Commun.

[CR128] Storer J, Hubley R, Rosen J, Wheeler TJ, Smit AF (2021). The Dfam community resource of transposable element families, sequence models, and genome annotations. Mob DNA.

[CR129] Suzuki MM, Bird A (2008). DNA methylation landscapes: provocative insights from epigenomics. Nat Rev Genet.

[CR130] Sylvester T, Hjelmen CE, Hanrahan SJ, Lenhart PA, Johnston JS, Blackmon H (2020) Lineage-specific patterns of chromosome evolution are the rule not the exception in Polyneoptera insects. Proc Biol Sci 287:20201388. 10.1098/rspb.2020.1388 10.1098/rspb.2020.1388 PMC7542826 32993470

[CR131] Talavera G, Lukhtanov VA, Rieppel L, Pierce NE, Vila R (2013). In the shadow of phylogenetic uncertainty: the recent diversification of Lysandra butterflies through chromosomal change. Mol Phylogenet Evol.

[CR132] Terry MD, Whiting MF (2005). Mantophasmatodea and phylogeny of the lower neopterous insects. Cladistics.

[CR133] The International Aphid Genomics Consortium (2010) Genome sequence of the pea aphid *Acyrthosiphon pisum*. PloS Biol 8:e1000313. 10.1371/journal.pbio.1000313 10.1371/journal.pbio.1000313 PMC2826372 20186266

[CR134] Tinghitella RM (2008). Rapid evolutionary change in a sexual signal: genetic control of the mutation ‘flatwing’ that renders male field crickets (*Teleogryllus oceanicus*) mute. Heredity.

[CR135] Tomioka K (2014). Chronobiology of crickets: a review. Zoolog Sci.

[CR136] Toxopeus J, Des Marteaux LE, Sinclair BJ (2019). How crickets become freeze tolerant: the transcriptomic underpinnings of acclimation in *Gryllus veletis*. Comp Biochem Physiol Part D Genomics Proteomics.

[CR137] Toxopeus J, McKinnon AH, Štětina T, Turnbull KF, Sinclair BJ (2019). Laboratory acclimation to autumn-like conditions induces freeze tolerance in the spring field cricket *Gryllus veletis* (Orthoptera: Gryllidae). J Insect Physiol.

[CR138] van Huis A, van Itterbeeck J, Klunder H, Mertens E, Halloran A, Muir G, Vantomme P (2013). Edible insects: future prospects for food and feed security.

[CR139] Wang X, Fang X, Yang P, Jiang X, Jiang F, Zhao D, Li B, Cui F, Wei J, Ma C, Wang Y, He J, Luo Y, Wang Z, Guo X, Guo W, Wang X, Zhang Y, Yang M, Hao S, Chen B, Ma Z, Yu D, Xiong Z, Zhu Y, Fan D, Han L, Wang B, Chen Y, Wang J, Yang L, Zhao W, Feng Y, Chen G, Lian J, Li Q, Huang Z, Yao X, Lv N, Zhang G, Li Y, Wang J, Wang J, Zhu B, Kang L (2014). The locust genome provides insight into swarm formation and long-distance flight. Nat Commun.

[CR140] Watanabe T, Ochiai H, Sakuma T, Horch HW, Hamaguchi N, Nakamura T, Bando T, Ohuchi H, Yamamoto T, Noji S, Mito T (2012). Non-transgenic genome modifications in a hemimetabolous insect using zinc-finger and TAL effector nucleases. Nat Commun.

[CR141] Whitfield JB, Kjer KM (2008). Ancient rapid radiations of insects: challenges for phylogenetic analysis. Annu Rev Entomol.

[CR142] Whitman DW (2008). The significance of body size in the Orthoptera: a review. J Orthoptera Res.

[CR143] Whittle CA, Kulkarni A, Extavour CG (2021). Evolutionary dynamics of sex-biased genes expressed in cricket brains and gonads. J Evol Biol.

[CR144] Whittle CA, Kulkarni A, Chung N, Extavour CG (2021). Adaptation of codon and amino acid use for translational functions in highly expressed cricket genes. BMC Genomics.

[CR145] Williams DM (2001) Largest. University of Florida Book of Insect Records, Chapter, p 30. https://entnemdept.ufl.edu/walker/ufbir/chapters/chapter_30.shtml. Accessed 25 Aug 2021

[CR146] Wipfler B, Letsch H, Frandsen PB, Kapli P, Mayer C, Bartel D, Buckley TR, Donath A, Edgerly-Rooks JS, Fujita M, Liu S, Machida R, Mashimo Y, Misof B, Niehuis O, Peters RS, Petersen M, Podsiadlowski L, Schütte K, Shimizu S, Uchifune T, Wilbrandt J, Yan E, Zhou X, Simon S (2019). Evolutionary history of Polyneoptera and its implications for our understanding of early winged insects. Proc Natl Acad Sci USA.

[CR147] Wu C, Lu J (2019). Diversification of transposable elements in arthropods and its impact on genome evolution. Genes (basel).

[CR148] Xu M, Shaw KL (2019) Genetic coupling of signal and preference facilitates sexual isolation during rapid speciation. Proc Biol Sci 286:20191607. 10.1098/rspb.2019.1607 10.1098/rspb.2019.1607 PMC6834049 31640515

[CR149] Xu M, Shaw KL (2021). Extensive linkage and genetic coupling of song and preference loci underlying rapid speciation in Laupala crickets. J Hered.

[CR150] Yang J, Dong H, He M, Gao J (2021). Mitochondrial genome characterization of *Gryllodes sigillatus* (Orthoptera: Gryllidae) and its phylogenetic implications. Mitochondrial DNA b: Resour.

[CR151] Ylla G, Nakamura T, Itoh T, Kajitani R, Toyoda A, Tomonari A, Bando T, Ishimaru Y, Watanabe T, Fuketa M, Matsuoka Y, Barnett AA, Noji S, Mito T, Extavour CG (2021). Insights into the genomic evolution of insects from cricket genomes. Commun Biol.

[CR152] Younger MA, Müller M, Tong A, Pym EC, Davis GW (2013). A presynaptic EnaC channel drives homeostatic plasticity. Neuron.

[CR153] Yuan H, Huang Y, Mao Y, Zhang N, Nie Y, Zhang X, Zhou Y, Mao S (2021) The evolutionary patterns of genome size in Ensifera (Insecta: Orthoptera). Front Genet 12:693541. 10.3389/fgene.2021.693541 10.3389/fgene.2021.693541 PMC8261143 34249107

[CR154] Zeng V, Ewen-Campen B, Horch HW, Roth S, Mito T, Extavour CG (2013) Developmental gene discovery in a hemimetabolous insect: *de novo* assembly and annotation of a transcriptome for the cricket *Gryllus bimaculatus*. PLoS One 8:e61479. 10.1371/journal.pone.0061479 10.1371/journal.pone.0061479 PMC3646015 23671567

[CR155] Zera AJ (2009) Wing polymorphism in Gryllus (Orthoptera:Gryllidae): Proximate endocrine, energetic and biochemical bases underlying morph specializations for flight vs. reproduction. In: Whiteman DW, Ananthakrishnan TN (eds) Phenotypic plasticity of insects: Mechanisms and Consequences: Science Publisher, Inc., New Hampshire, pp 609–653

[CR156] Zera AJ, Vellichirammal NN, Brisson JA (2018). Diurnal and developmental differences in gene expression between adult dispersing and flightless morphs of the wing polymorphic cricket, *Gryllus firmus*: Implications for life-history evolution. J Insect Physiol.

[CR157] Zhang X, Rayner JG, Blaxter M, Bailey NW (2021). Rapid parallel adaptation despite gene flow in silent crickets. Nat Commun.

[CR158] Zhou Z, Zhao L, Liu N, Guo H, Guan B, Di J, Shi F (2017). Towards a higher-level Ensifera phylogeny inferred from mitogenome sequences. Mol Phylogenet Evol.

[CR159] Zsögön A, Čermák T, Naves ER, Notini MM, Edel KH, Weinl S, Freschi L, Voytas DF, Kudla J, Peres LEP (2018). *De novo* domestication of wild tomato using genome editing. Nat Biotechnol.

[CR160] Zuk M, Rotenberry JT, Tinghitella RM (2006). Silent night: adaptive disappearance of a sexual signal in a parasitized population of field crickets. Biol Let.

